# Modelling the Impact of Condom Distribution on the Incidence and Prevalence of Sexually Transmitted Infections in an Adult Male Prison System

**DOI:** 10.1371/journal.pone.0144869

**Published:** 2015-12-14

**Authors:** Nick Scott, Emma McBryde, Amy Kirwan, Mark Stoové

**Affiliations:** 1 Centre for Population Health, Burnet Institute, Melbourne, VIC 3004, Australia; 2 Department of Epidemiology and Preventive Medicine, Monash University, Clayton, VIC 3008, Australia; 3 Australian Institute of Tropical Health and Medicine, James Cook University, Townsville, QLD 4811, Australia; 4 Department of Medicine, The University of Melbourne, Parkville, VIC 3050, Australia; University of Illinois at Urbana-Champaign, UNITED STATES

## Abstract

**Aims:**

To determine the effects of 1) a condom distribution program and 2) a condom distribution program combined with opt-out sexually transmitted infection (STI) screening on the transmission and prevalence of STIs in a prison system.

**Methods:**

Using data from an implementation evaluation of a state-wide prison condom program and parameter estimates from available literature, a deterministic model was developed to quantify the incidence and prevalence of sexually transmitted HIV, hepatitis B, chlamydia, syphilis and gonorrhoea across 14 Victorian prisons. The model included individual prison populations (by longer (>2 years) or shorter sentence lengths) and monthly prisoner transfers. For each STI, simulations were compared: without any intervention; with a condom distribution program; and with a combined condom and opt-out STI screening at prison reception intervention program.

**Results:**

Condoms reduced the annual incidence of syphilis by 99% (N = 66 averted cases); gonorrhoea by 98% (N = 113 cases); hepatitis B by 71% (N = 5 cases); chlamydia by 27% (N = 196 cases); and HIV by 50% (N = 2 cases every 10 years). Condom availability changed the in-prison epidemiology of gonorrhoea and syphilis from self-sustaining to levels unlikely to result in infection outbreaks; however, condoms did not reduce chlamydia prevalence below a self-sustaining level due to its high infectiousness, high prevalence and low detection rate. When combined with a screening intervention program, condoms reduced chlamydia prevalence further, but not below a self-sustaining level. The low prevalence of HIV and hepatitis B in Australian prisons meant the effects of condoms were predicted to be small.

**Conclusion:**

Condoms are predicted to effectively reduce the incidence of STIs in prison and are predicted to control syphilis and gonorrhoea transmission, however even combined with a screening on arrival program may be insufficient to reduce chlamydia prevalence below self-sustaining levels. To control chlamydia transmission additional screening of the existing prison population would be required.

## Introduction

Prisoners experience markedly poorer physical and mental health and elevated risk of disease compared to the general community, often compounded by entrenched disadvantage and drug dependence [1–3]. Among prisoners in Australia there is a high prevalence of hepatitis C and hepatitis B [4], and although the prevalence of HIV is estimated to be low compared to other developed nations’ prisons, it is still greater than in the general Australian community [5]. While a self-reported history of one or more sexually transmitted infections (STIs) is common among prisoners [6, 7], estimates of the biological prevalence of STIs in prison is less clear. STI testing rarely occurs for males despite the provision of voluntary testing offered at both original reception to prison and upon subsequent transfers [8]. Further, the limited biological prevalence studies show inconsistent findings: a chlamydia surveillance programme in New South Wales (NSW) prisons between 2005 and 2007 found a chlamydia notification rate approximately four times higher than the general community [9]; however national surveillance among a limited number of prisoners across Australian jurisdictions showed rates of STIs similar to the general Australian population [4].

Despite paucity of information regarding the rate and burden of disease associated with STIs in Australian prisons, there is evidence on behaviours amongst prisoners that suggest the prison setting itself is a high risk environment for STI transmission between prisoners and potentially to the general community following prisoner release. Studies of prisoner risk behaviour in Victoria and NSW demonstrated continued risk for blood borne virus (BBV) and STI transmission through unprotected sex and injecting and tattooing with unsterile equipment [4, 5, 10–15], alongside widespread reports among prisoners of sexual activity in prisons without condoms being available [16]. Closed settings where sexual risk behaviours are known to occur provide ideal settings for STI outbreaks should undetected (and untreated) infections enter the susceptible population [17, 18], with outbreaks of syphilis, gonorrhoea and hepatitis B being reported in prisons previously [19]. Australia’s Third National Sexually Transmissible Infections Strategy 2014–2017 [20] includes people in custodial settings as a high-risk priority population for the prevention of STIs, noting that prisoners often have limited access to STI education and prevention tools prior to incarceration and that this is compounded by similar limited access in prison.

Condoms are an effective public health and harm minimisation measure to reduce transmission of STIs and some BBVs [21]. The restricted movements of prisoners and associated stigma make it unrealistic to expect 100% efficacy from a condom distribution program and so further intervention will likely be required to prevent outbreaks. An opt-out STI screening program on arrival to prison for STIs may offer an additional opportunity to eliminate treatable STIs such as chlamydia, gonorrhoea and syphilis and thus prevent onward transmission. Similar opt-out screening programs in community settings have been shown to be successful in dramatically increasing rates of screening and treatment [22].

In this paper we use data from an implementation evaluation of a jurisdiction-wide prison condom program and parameter estimates from available literature to report on models estimating changes to the epidemiology of STIs that are likely to occur subsequent to: 1) the introduction of a condom distribution program in a prison system; and 2) the introduction of condoms, in conjunction with an opt-out screening on arrival strategy.

## Methods

A deterministic (equation based) model was used to quantify the incidence and prevalence of sexually transmitted HIV, hepatitis B, syphilis, chlamydia and gonorrhoea in all 14 adult male prisons in Victoria.

### Setting

Victoria is the second most populous state in Australia and has 14 prisons, 12 publically operated and 2 privately operated. All Victorian prisons are managed by Corrections Victoria and overseen by the Victorian Department of Justice. At June 30th 2013, Victoria had a male incarceration rate of 227 per 100,000 males per year and a total male prison population of 4,964 (representing 93% of the total prison population). The median age of a Victorian prisoner was 35.3 years, 7% were indigenous (the lowest of all states and territories in Australia), and 25% were born overseas, most commonly in Vietnam. Prisoners were incarcerated for offences classified as: acts intended to cause injury (15.9%); offences against justice procedures, government security and operations (14.9%); sexual assault (12.7%); illicit drugs (11.9%); unlawful entry with intent (10.3%); homicide (8.9%); and robbery and extortion (8.4%). Further details are available from the Australian Bureau of Statistics [23].

In Australia, condoms are currently available in prisons in the Australian Capital Territory and the states of New South Wales, South Australia and Western Australia [12, 24]; however prior to the condom distribution program considered in this analysis, no condoms were available to prisoners in Victoria.

### Data sources

For the period July 2012 to June 2013 monthly prison populations, receptions, discharges and transfers were obtained from Corrections Victoria for 14 male prisons, decomposed as either long (more than two years) or short sentences. For each prison (i = 1,…,14), the monthly averages for prison population (P_i_), proportion of prisoners serving short sentences, number of short and long sentence discharges (Ns_i_ and Nl_i_ respectively), number of transfers between each of the other 13 prisons, and number of condoms distributed (Dist_i_) were calculated and used to define populations, movements and turnovers within the model. Across prisons, the median prison population was 332 (inter quartile range (IQR) 223–396), the median proportion of prisoners serving a short sentence was 0.5 (IQR 0.38–0.62), the median short sentence and long sentence discharge rates were 16.4% (IQR 11.5–22.1%) per month and 2.0% (IQR 1.1–2.9%) per month respectively, and the median number of condoms distributed in each prison was 65 (IQR 24–98) per month.

Estimates from the literature were found for: the proportion of prisoners who are sexually active (Sex), the proportion of distributed condoms used for sex (Used), the proportion of total sexual acts that would use condoms when available (Condom), the prevalence of each STI in the community (Cinf), hepatitis B community vaccination rate (VacC), hepatitis B prison population vaccination rate (VacP), the proportion of the prison population with prior incarceration (Prior), STI transmission risks per sexual act (β), STI incubation periods (γ), STI detection window periods (ω), STI detection and treatment rates (τ), and STI treatment lengths (η). A description of parameters and their sources is provided in Table 1.

**Table 1:** Parameters used to model sexual activity, STI infection rates, STI transmission and treatment rates and STI screening rates in Victorian prisons.

Parameter	Description	Breakdown	Value	Source
**Sexual activity**				
Sex	Proportion prisoners sexually active		0.09	Butler et al. 2010^a^ [25]
Used	Proportion of distributed condoms used for sex		0.40	Dolan et al. 2004 [12]
Condom	Proportion of sexual acts using condoms (when available)		0.52	Dolan et al. 2004 [12]
Acts	Number of sex acts per month for each sexually active prisoner^b^.		$\frac{Dist\times Used}{P\times Sex\times Condom}$	Estimation of sexual activity based on the number of condoms distributed, and estimations of the proportion used for sex and proportion of sexually active individuals.
**Infection rates**				
Cinf	Community prevalence *(closest estimates to prison demographic*: *interquartile age range 25–45 years*, *93% male 7% female)*	HIV	0.2%	UNAIDS [26]
		Hepatitis B	1.7% (25–39 year olds)	Butler et al. 2011 [4]
		Syphilis	0.26% (men)	WHO 2005^c^ [27]
		Chlamydia	3.9% (men <30)	Lewis et al. 2012 [28]
		Gonorrhoea	0.52% (men)	WHO 2005^c^ [27]
VacHB	Hepatitis B vaccination rate	Community	30%	Expert opinion
		Prisoners	50% (males, 2010)	Butler et al. 2011 [4]
Prior	Proportion of new prisoners with prior incarceration		0.48	Department of Justice
Pinf	Current prison prevalence		$\frac{I+L+T+V}{P}$	Infected proportion of prisoners in the model at any point in time.
**Transmission and treatment rates**				
β	Risk of transmission per sex act ^d^	HIV	0.014	Baggaley et al. 2010 [29]
β	“	Hepatitis B	0.07	Expert opinion
β	“	Syphilis	0.15	Wilson et al. 2010 [30]
β	“	Chlamydia	0.35	Gray et al. 2009 [31]
β	“	Gonorrhoea	0.22	Wilson et. al. 2010 [30]
λ	Risk of transmission per month		β x (1-Condom) x Acts x (proportion infectious sexually active prisoners)	Likelihood of becoming infected each month for a given level of sexual activity, transmission risk per act, probability of using a condom each time and current infection rate among possible partners. Prisoners who are infected and on treatment are assumed to be infectious for all STIs except HIV, where antiretroviral therapy is assumed to supress the virus enough that transmission is negligible [32].
τ	Effective detection and treatment rate^e^	HIV	1/18	The Kirby Institute 2014 [33]; using estimated time from initial infection to a CD4 count of 432—the reported Australian average on diagnosis—and the guidelines to treat at any CD4 count in Australia [34, 35].
τ	“	Hepatitis B	1/12	Expert opinion
τ	“	Syphilis	1/2	Expert opinion
τ	“	Chlamydia	1/12	Expert opinion
τ	“	Gonorrhoea	1	Expert opinion
η	Effective treatment length	HIV	Lifetime	Expert opinion
η	“	Hepatitis B	6 months (90%); Lifetime (10%)	WHO [27]
η	“	Syphilis	1 week	Melbourne sexual health centre 2012 [36]
η	“	Chlamydia	1 week	Melbourne sexual health centre 2012 [36]
η	“	Gonorrhoea	1 week	Melbourne sexual health centre 2012 [36]
γ	Duration of latency	HIV	1 week	WHO 2015 [34]
γ	“	Hepatitis B	6 months	WHO 2005 [27]
γ	“	Syphilis	3 weeks	Heymann 2008 [37]
γ	“	Chlamydia	1.5 weeks	Heymann 2008 [37]
γ	“	Gonorrhoea	1 week	Heymann 2008 [37]
ω	Detection window period	HIV	3 weeks	Rosenberg et al. 2015 [38]
ω	“	Hepatitis B	4 weeks	Expert opinion
ω	“	Syphilis	2.5 weeks	Expert opinion
ω	“	Chlamydia	1 week	Expert opinion
ω	“	Gonorrhoea	5 days	Expert opinion
**Screening intervention**				
ρ	Screening rate		1 month	Assumed
screen	Proportion who are screened		95%	Assumed

^a^ No evidence was available to support the stratification *Sex* by sentence length.

^b^ A minimum of one sexual act per month is assumed for sexually active prisoners.

^c^ Western Pacific region (men).

^d^ The transmission risk associated with a single type of sexual contact is assumed for each STI: HIV is for unprotected anal; HBV is estimated as 5 times that of HIV; syphilis, chlamydia and gonorrhoea are for unprotected vaginal (unprotected anal estimates were unavailable).

^e^Includes spontaneous clearance of STIs (except HIV) in the absence of treatment.

### Model description

Independent models were used for the sexual transmission of HIV, hepatitis B, syphilis, chlamydia and gonorrhoea, as described below. Complete equations for each of these models are provided in the supporting information (S1 File).

#### Single prison model, HIV, syphilis, chlamydia and gonorrhoea

Prisoners were classified as either: U—not sexually active in prison and uninfected; V—not sexually active in prison and infected; S—sexually active in prison and not infected; L—sexually active in prison and infected, with disease in latent period; I—sexually active in prison and infected; or T—sexually active in prison and in treatment for a current infection.

**Fig 1:**
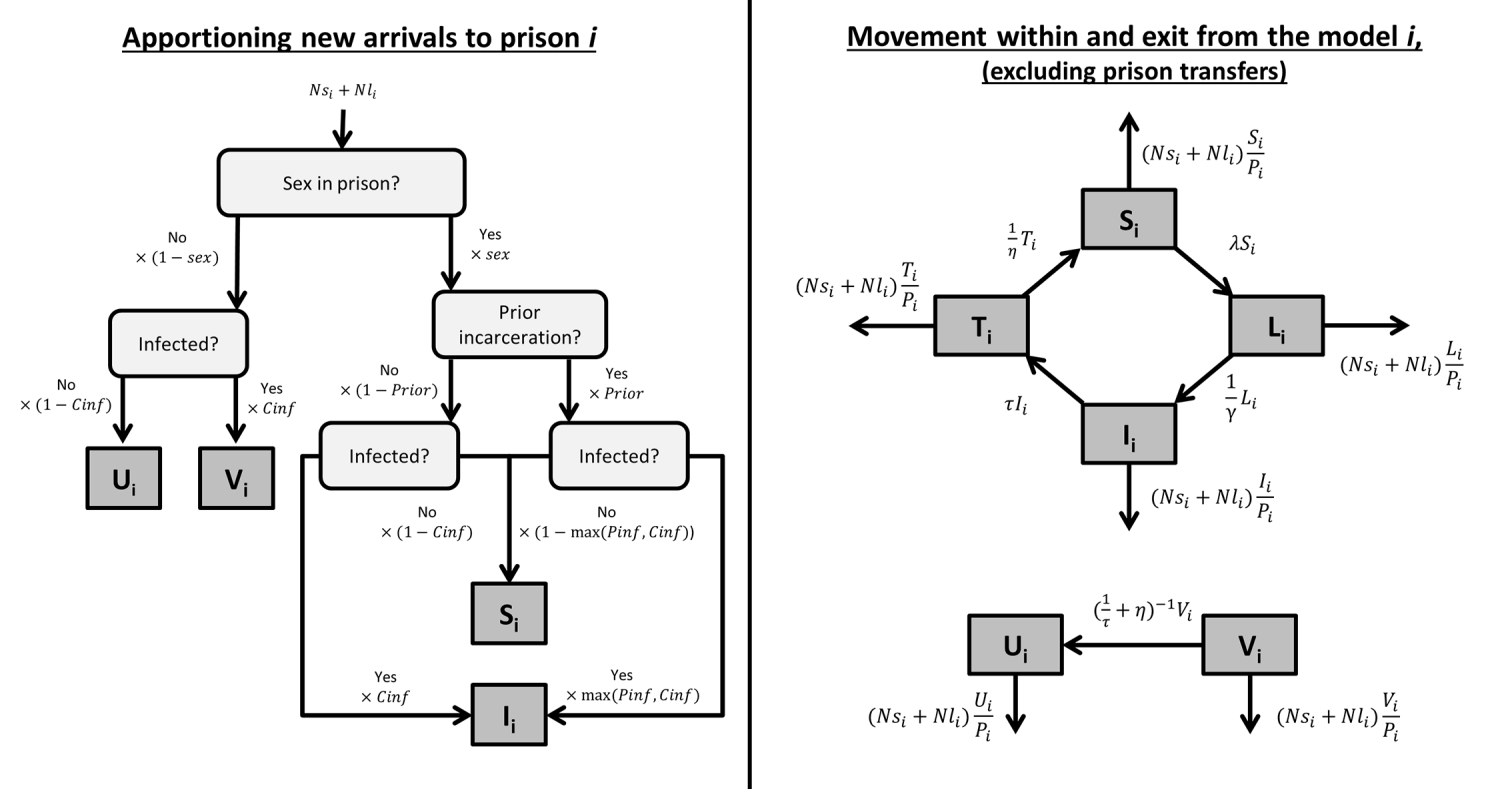
Single prison HIV, syphilis, chlamydia and gonorrhoea model. Each month Ns_i_+Nl_i_ new prisoners arrived in prison i and were apportioned to compartments according to sexual activity in prison, prior incarceration status and community or prison infection prevalence (left). Prisoners could then move between compartments in the model or leave if discharged (right).

New receptions to prison i (Ns_i_ + Nl_i_ prisoners each month) were apportioned into the U_i_, V_i_, S_i_ and I_i_ compartments according to whether or not they were likely to: be sexually active in prison (Sex); have had prior incarceration (Prior); be infected based on community infection prevalence if they had no history of incarceration (Cinf); and be infected based on the greater of current prison infection prevalence or community infection prevalence if they had a history of incarceration (Pinf), as shown in Fig 1, left panel. The prevalence of infection among new arrivals with a history of incarceration was selected in this way to represent both the additional STI exposure of these prisoners, and their socio-economic disadvantage in the community. During each month: λS_i_ susceptible prisoners became infected with the latent disease (where λ is the force of infection defined in Table 1); (1/γ)L_i_ prisoners moved from the latent phase to infection phase; τI_i_ infected prisoners undertook treatment for the disease; (1/η)T_i_ prisoners were successfully treated; and infected but not sexually active prisoners were detected and treated at a rate of ((1/τ)+η)^-1^. Discharged prisoners were assumed to come with equal probability from each compartment, at rates balanced to maintain a constant prison population (Fig 1, right panel).

#### Single prison model, hepatitis B

The model for hepatitis B differed due to the existence of vaccinations and immunity conferred through past exposure. For this model, new arrivals were apportioned with the additional consideration that if they were vaccinated or had past expose (according to community vaccination rates (VacC) or prison vaccination rates (VacP) for the proportion with prior incarceration) they were moved into the U compartment regardless of sexual activity (see S1 Fig). The number of sexual acts per month per sexually active prisoner was also reduced by a factor [Prior*(1-VacP)+(1-Prior)*(1-VaccC)], which is the probability of involving an unvaccinated prisoner (an additional requirement for infection in this model).

#### Prisoner transfers

The above models were applied to each of the 14 prisons, which were then linked by a prisoner transfer matrix, allowing movements between prisons. Transfers were assumed to be independent of sexual activity and STI status. Details are provided in the supporting information (S1 File).

#### Screening on arrival

**Fig 2:**
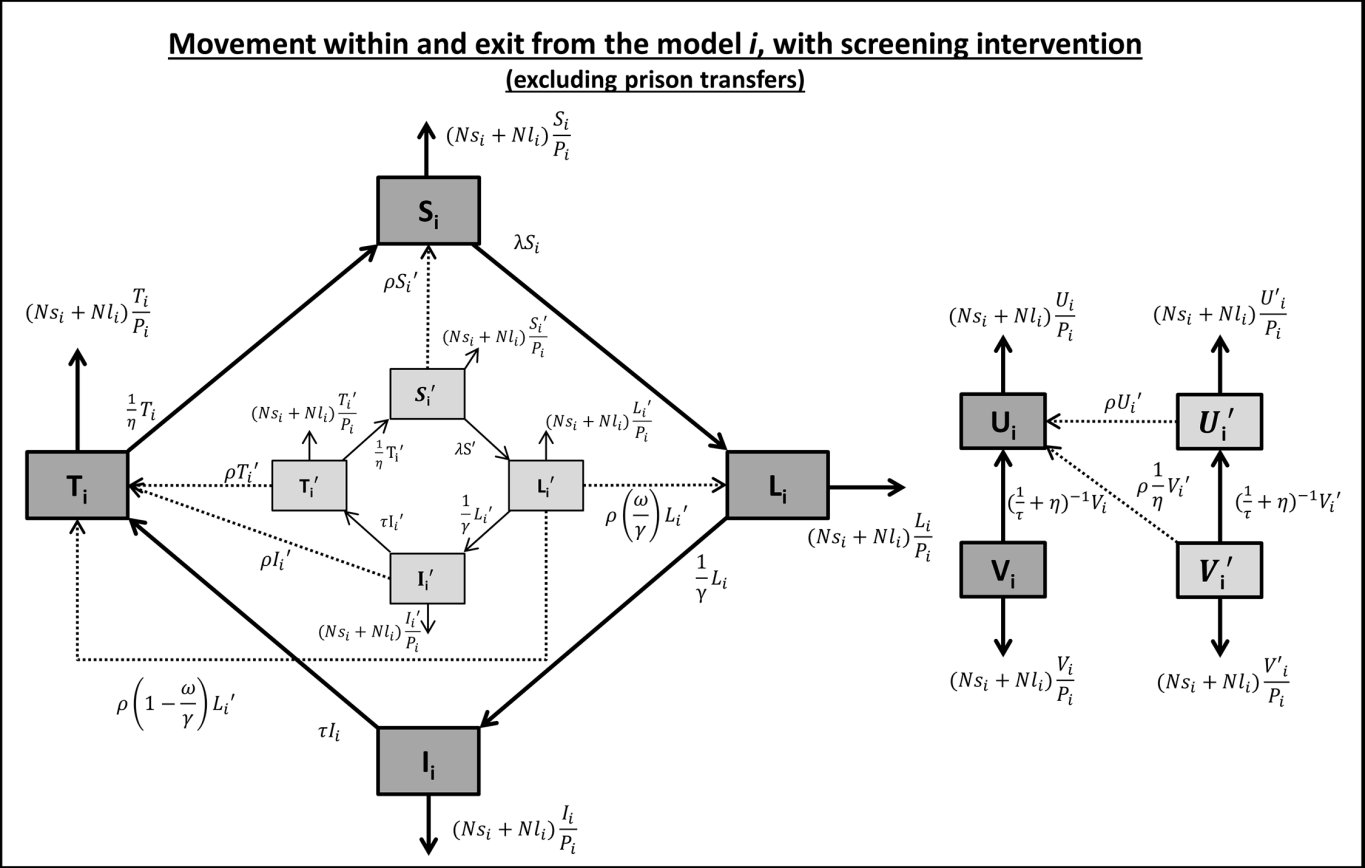
Single prison HIV, syphilis, chlamydia and gonorrhoea model with screening on arrival. After new arrivals entered the ‘pre-screen’ (lighter) compartments, their movements mirrored the original (darker) compartments where they moved to after they were screened.

To capture the effects of a STI screening on arrival initiative, the models were expanded to include six additional ‘pre-screen’ compartments (U’, S’, L’, I’, T’ and V’), with dynamics mirroring the original ones. A proportion (screen) of new arrivals—including from a prison transfer—entered the model into the pre-screen compartments where they could still interact as before with the entire population. They were then screened (at an estimated screening rate ρ) and moved into the original compartments: those who were susceptible, in treatment or infected moved to the original susceptible, treatment or treatment compartments respectively, while those who had latent disease moved to the original treatment compartment if they had passed their detection window, or to the original latent compartment if they had not (Fig 2). Discharges and transfers were assumed to come with equal probability from each of the 12 compartments, and for the hepatitis B model, a screening program was considered to include vaccination, so that the S’ compartment flowed into the U compartment instead of S.

### Model uncertainty

The literature around the sexual behaviours and STI prevalence among prisoners is sparse, and studies often experience low response rates and struggle to obtain accurate parameter estimates. To test model robustness, a Monte Carlo uncertainty analysis was conducted. Here we defined the uncertainties of individual parameters, parameterised this uncertainty as a probability distribution of each parameter and undertook multiple simulations using random, independent parameter draws. We developed 95% credible intervals (95%CrI) for the resulting STI prevalence and reduction in incidence after each intervention by taking the central 95% of outcomes (i.e. the 2.5–97.5 percentiles) of 2000 alternate runs. Beta distributions were used to estimate the uncertainty around the parameters Sex, Used, Condom, Prior, ρ, screen, VacHB, Cinf, β, τ, γ and ω. The distributions used for each parameter are provided in S1 Table, and plotted in S2 Fig and S3 Fig.

## Results

### Prevalence and incidence

**Fig 3:**
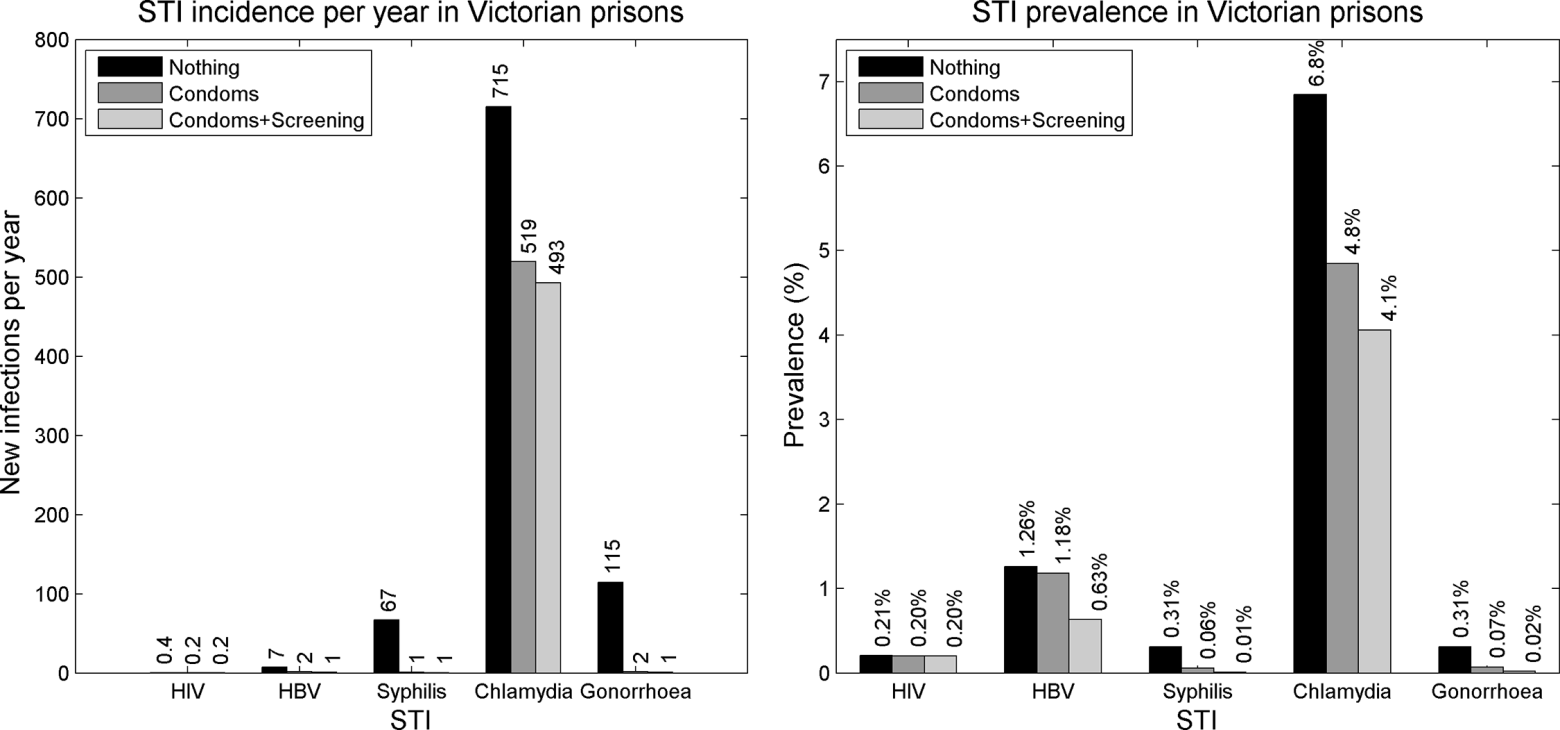
Total incidence and prevalence. Estimated incident infections per annum from unsafe sex (left) and in-prison prevalence of STIs (right) with no intervention, after condoms were made available and with a combined condoms and a screening on arrival intervention.

The model predicts that the availability of condoms alone would reduce the annual number of sexually acquired cases of: hepatitis B by 71% (N = 5 averted cases); syphilis by 99% (N = 66 averted cases); chlamydia by 27% (N = 196 averted cases); gonorrhoea by 98% (N = 113 averted cases); and HIV by 50% (N = 2 averted cases every 10 years). Augmenting this with a screening on arrival program led to a further 15% reduction in incident cases of hepatitis B (N = 1 additional case from condoms alone), a further 4% reduction in incident cases of chlamydia (N = 26 additional cases from condoms alone), a further 1% reduction in incident cases of gonorrhoea (N = 1 additional case from condoms alone) and had no effect on HIV and syphilis incidence (Fig 3).

Condoms were able to virtually control the transmission of syphilis and gonorrhoea. Despite preventing a number of new hepatitis B infections, the availability of condoms did not stop low level transmission. The addition of a screening intervention to condom distribution made little impact on the number of averted hepatitis B, syphilis and gonorrhoea infections per year, however it led to further reductions hepatitis B, chlamydia, syphilis and gonorrhoea prevalence. HIV prevalence was so low that the effects of condoms and screening were small.

### Uncertainty analysis

Outcomes produced by point estimates using parameters from Table 1 were very close to median values from the sensitivity analysis (Table 2) and appear reasonable estimates of the prevalence and prevented infections in each scenario. The number of averted infections per year when condoms were introduced was slightly right skewed for HIV, hepatitis B and chlamydia with medians 0.17, 5 and 172 respectively, while syphilis and gonorrhoea both had high proportions of outcomes with fewer than 50 infections per year prevented and long tails extending to some realisations near 600 (Table 2, S4 Fig).

**Table 2:** Comparison of model outcomes for STI prevalence and infections averted using point estimates to those from a Monte Carlo uncertainty analysis.

	Point Estimate			Sensitivity analysis median (2.5,97.5 percentile)		
	No intervention	Condoms	Condoms + screen	No intervention	Condoms	Condoms + screen
**Prevalence**						
HIV	0.21%	0.20%	0.20%	0.19% (0.01–4.79)	0.19% (0.07–0.40)	0.19% (0.07–0.40)
Hepatitis B	1.26%	1.18%	0.63%	1.16% (0.34–2.68)	1.07% (0.31–2.51)	0.59% (0.17–1.46)
Syphilis*	0.31%	0.06%	0.01%	0.27% (0.01–2.04)	0.06% (0.01–0.18)	0.01% (0.00–0.19)
Chlamydia	6.8%	4.8%	4.1%	9.0% (6.8–11.0)	7.2% (4.2–9.5)	4.4% (2.3–6.1)
Gonorrhoea*	0.30%	0.07%	0.02%	0.30% (0.04–1.60)	0.07% (0.03–0.14)	0.02% (0.01–0.10)
**Infections averted per year**						
HIV		0.2	0.2		0.17 (0.01–4.79)	0.17 (0.01–4.79)
Hepatitis B		5	6		5 (1–16)	6 (1–19)
Syphilis*		66	67		54 (0–479)	55 (0–466)
Chlamydia		196	222		172 (62–489)	193 (0–523)
Gonorrhoea*		113	114		108 (1–677)	109 (1–681)

*Due to the large right skew of infections prevented and prevalence, the 0–95 percentiles have been used as the 95%CrI.

## Discussion

These models have demonstrated that as each of the STIs considered are in different epidemic stages within the prison system different responses are required.

For syphilis and gonorrhoea, condoms were able to eliminate the possibility of self-sustaining outbreaks and virtually eliminate new infections from the system. For these infections, the basic reproduction ratio (R0)—the expected number of new infections that occur when a typical infected case arrives in a fully susceptible population (R0 = Acts*(1-Condom)*β/τ, [39])—was less than but close to one at default parameters, and above one for some part of the parameter space sampled in the uncertainty analysis (as evidenced by the large 95%CrIs in Table 2). In general, a preventative intervention for an infectious disease will be most effective if it can cause a change in R0 from above one to below one. For syphilis and gonorrhoea, condoms were able to achieve this for the majority of the simulations (see S4 and S5 Figs). Further, if R0 is close to exceeding R0 = 1, sporadic outbreaks may be seen in the prison system and, once introduced, these infections may become self-sustaining. By shifting R0 away from this threshold condom use has made outbreaks of syphilis and gonorrhoea such as those previously seen internationally [40–42] significantly less likely.

The model predicts that chlamydia is self-sustaining within the prison system, and due its higher prevalence and transmissibility, condoms, a screening on arrival program *and* testing and treatment of the existing prison population would be required in order to completely control the possibility of outbreaks. Compared to syphilis and gonorrhoea, the higher transmissibility and longer detection times (due to more asymptomatic cases [43]) results in R0 being above one for chlamydia, even when condoms are available. Thus, despite being successful at reducing incidence, condom use was not sufficient to control transmission, and although further reducing incidence, combining condom use with screening on arrival was also insufficient to control transmission. This is particularly problematic, given that the data available suggests that chlamydia is by far the most prevalent STI in Victorian prisons and in the Australian community [28].

Despite preventing a number of hepatitis B transmissions, availability of condoms will not stop low level transmission of hepatitis B, as a result of long treatment times and the possibility of chronic infection. However if condom availability were combined with vaccination of all prisoners on arrival, the transmission of hepatitis B could be completely controlled. Similarly, the prevalence of HIV is so low in Victorian prisons that the effects of condoms are predicted to be small. This would not be the case in many international settings, where HIV prevalence in prison can exceed 10% [44]. Nevertheless, the high and ongoing costs associated with treating HIV and chronic hepatitis B mean that even a few averted cases are likely to have significant cost benefits.

The importance of condoms to prevent STI transmission have been known for some time [21]. In particular, condoms have already been made available in some European, Canadian and Australian (excluding Victorian) prisons, where they have been found to be effective, with no evidence of condom availability resulting in increases in prison sexual activity, sexual assaults or other adverse incidents [11, 12, 24, 45–47]. Given the clear benefits highlighted by these models and the lack of negative consequences reported from distribution programs elsewhere, the provision of condoms to such a highly vulnerable population is a simple harm reduction initiative that falls under a basic duty of care, offering some level of protection both to prisoners and the community upon their release.

This paper has several limitations. First, information about the health and sexual behaviour of prisoners is difficult to obtain, and these models would benefit from more information on the prevalence of STIs on arrival and many of the other parameter estimates used. Such data would allow better calibration and improve accuracy and predictive power. Second, the assumption that 9% of prisoners were sexually active may be an under-estimate as it is based on self-reported data from Butler et al. [25]. However conversely, the risk of STI transmission between prisoners in the model may be over-estimated, since the model has assumed a single transmission risk associated with a single type of sexual contact for each STI; in the Butler et al. study prisoners reported different forms of sexual contact that have varying STI transmission risks—for example the risk of HIV transmission through oral sex is minimal compared to the risk through unprotected anal intercourse. A single transmission risk was used in the model as a result of the limited data available to separate sexual activity by type, and the limited studies estimating the transmission probability per sexual act for different types of sexual contact. We have instead attempted to capture the effects of different sexual contact types by varying the average transmission probabilities in the uncertainty analysis. Third, the models have assumed no interaction between STIs, and that transmission occurs only through sexual contact (i.e. no hepatitis B or HIV transmission through needle sharing). These are conservative assumptions and may understate prevalence, as for example, people with HIV are more likely to develop chronic hepatitis B [48] and are more susceptible to other STIs, meaning that where transmission through other mediums is possible the likelihood of sexual transmission is also higher. This explains why for hepatitis B and gonorrhoea the prevalence in prison is slightly lower than the estimates of community prevalence, and we emphasize that the model has only been used to calculate relative changes in STI epidemiology as a result of changes to sexual transmission. Fourth, deterministic models assume perfect mixing of sexual partners within each prison regardless of sentence length or prior incarceration status; however there are few studies monitoring sexual networks in prisons meaning that data is limited and unlikely to be accurate enough to inform network models.

## Conclusions

The introduction of condoms in Victorian prisons is predicted to avert a number of STI transmissions. The model estimates that the availability of condoms could reduce the annual incidence syphilis by 99% (N = 66 averted cases); gonorrhoea by 98% (N = 113 cases); sexually acquired hepatitis B by 71% (N = 5 cases); chlamydia by 27% (N = 196 cases); and sexually acquired HIV by 50% (N = 2 cases every 10 years). The model predicted that augmenting a condom distribution program with a screening on arrival program would provide only modest additional gains, further reducing the annual incidence of gonorrhoea by 1% (N = 1 case), sexually acquired hepatitis B by 15% (N = 1 case), chlamydia by 4% (N = 26 cases), and would provide no additional reduction to syphilis and HIV incidence.

In relation to the *control* of STI transmissions in prison, a condom distribution program is predicted to have varying success. For syphilis and gonorrhoea, condoms are predicted to virtually eliminate new infections and the possibility of self-sustaining outbreaks; however, even combined with a screening on arrival program, condoms were insufficient to reduce chlamydia prevalence below self-sustaining levels. To control chlamydia transmission, additional screening of the existing prison population would be required. The prevalence of HIV and hepatitis B is so low that the effects of condoms are predicted to be small, however the high and ongoing costs associated with treatment means that even a few averted cases are likely to have significant cost benefits.

## Supporting Information

S1 Fig Entry into the hepatitis B model. For the hepatitis B model, each month Ns_i_+Nl_i_ new prisoners arrive in prison i and are apportioned to compartments according to sexual activity in prison, prior incarceration status vaccination status and community or prison infection prevalence.
(TIF)

S2 Fig Uncertainty distributions for non-STI specific parameters and hepatitis B vaccination coverage parameters. Assumed uncertainty of parameters for: the proportion of prisoners who are sexually active (Sex); the proportion of condoms used for sex (Used); the proportion of sexual acts that use condoms when available (Condom); the proportion of prisoners with a history of incarceration (Prior); the proportion of prisoners who are screened on arrival when the intervention is available and the rate they are screened at (in months); and the prevalence of hepatitis B vaccination in the community [VacHB (Community)] and in prison [VacHV (Prison)].
(TIF)

S3 Fig Uncertainty distributions for STI specific parameters. Assumed uncertainty of parameters for the community STI prevalence (proportion of community infected), the risk of transmission per sexual act, the effective detection and treatment rate (in months), the duration of latency (in months) and the window period (in months).
(TIF)

S4 Fig Uncertainty analysis infections averted. Histograms of HIV, Hepatitis B (HBV), syphilis (SYP), chlamydia (CHL) and gonorrhoea (GON) infections prevented per annum from 2000 simulations using random parameter draws, condom intervention (left) and condom with screening on arrival intervention (right).
(TIF)

S5 Fig Uncertainty analysis prevalence results. Histograms of HIV, Hepatitis B (HBV), syphilis (SYP), chlamydia (CHL) and gonorrhoea (GON) prevalence in prison using 2000 random parameter draws, before any interventions (left), after the introduction of condoms (middle), and after the introduction of condoms and a screening on arrival intervention (right).
(TIF)

S1 File Model equations, new arrivals in the hepatitis B model and the uncertainty analysis. Detailed description of the equations used for each model; the apportioning of new arrivals in the hepatitis B model, dealing with immunity through the vaccination; and the distributions used for individual parameter uncertainties, the resulting distributions of number of infections prevented per year (with condoms and with condoms + screening on arrival), and the resulting distributions of the prevalence of each infection (without condoms, with condoms and with condoms + screening on arrival).
(DOCX)

S1 Table Parameter uncertainty distributions. The uncertainties of individual parameters were parameterised as Beta probability distributions with parameters b_1_ and b_2._
(DOCX)
