# Supplementary figures and images for: Modelling the Impact of Condom Distribution on the Incidence and Prevalence of Sexually Transmitted Infections in an Adult Male Prison System
Source: PLoS One. 2015 Dec 14;10(12):e0144869. doi: 10.1371/journal.pone.0144869 (PMC4691199; doi:10.1371/journal.pone.0144869)

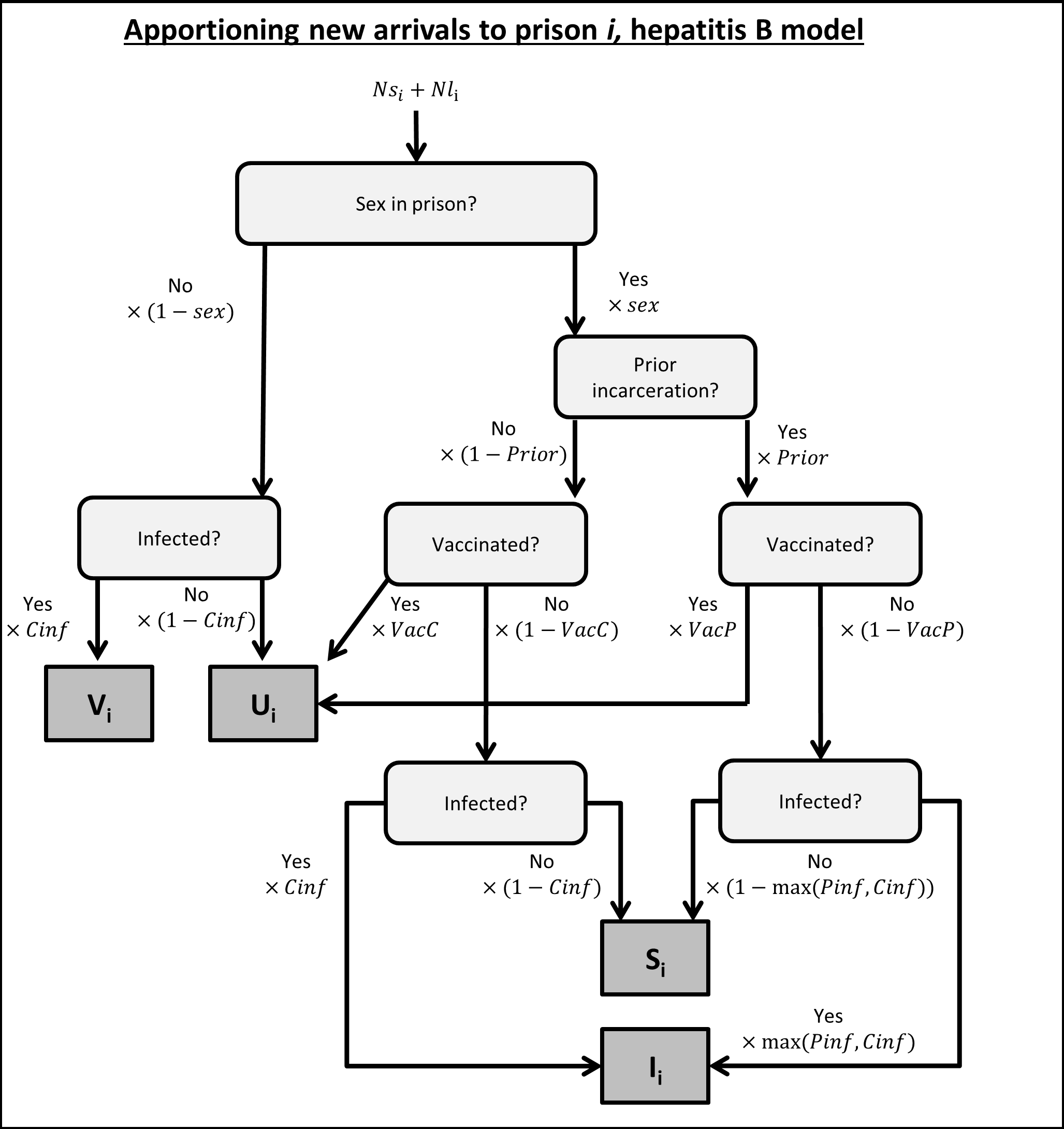

Supplement: S1 Fig — For the hepatitis B model, each month Nsi+Nli new prisoners arrive in prison i and are apportioned to compartments according to sexual activity in prison, prior incarceration status vaccination status and community or prison infection prevalence. (TIF) [file pone.0144869.s001.tif]

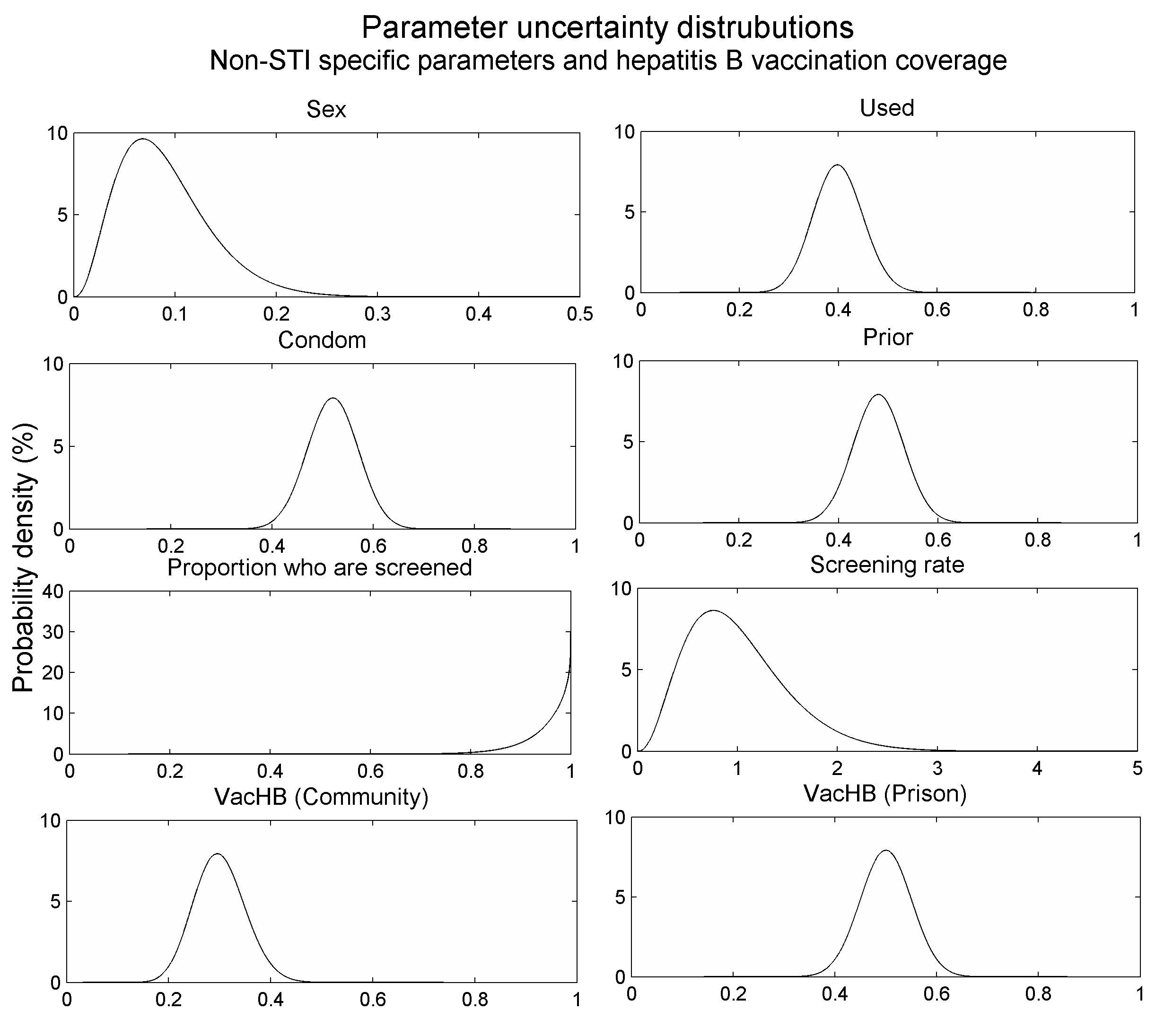

Supplement: S2 Fig — Assumed uncertainty of parameters for: the proportion of prisoners who are sexually active (Sex); the proportion of condoms used for sex (Used); the proportion of sexual acts that use condoms when available (Condom); the proportion of prisoners with a history of incarceration (Prior); the proportion of prisoners who are screened on arrival when the intervention is available and the rate they are screened at (in months); and the prevalence of hepatitis B vaccination in the community [VacHB (Community)] and in prison [VacHV (Prison)]. (TIF) [file pone.0144869.s002.tif]

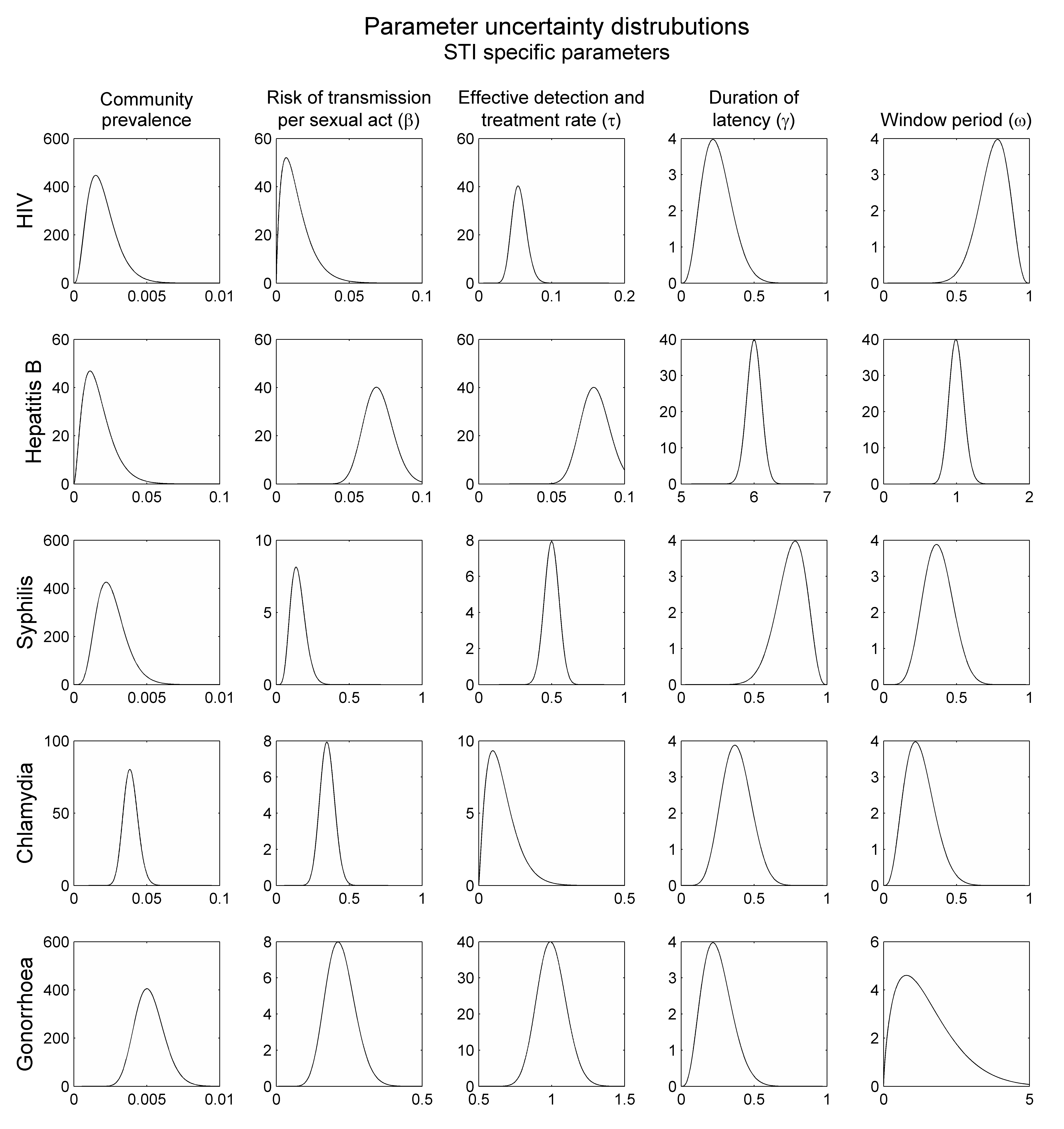

Supplement: S3 Fig — Assumed uncertainty of parameters for the community STI prevalence (proportion of community infected), the risk of transmission per sexual act, the effective detection and treatment rate (in months), the duration of latency (in months) and the window period (in months). (TIF) [file pone.0144869.s003.tif]

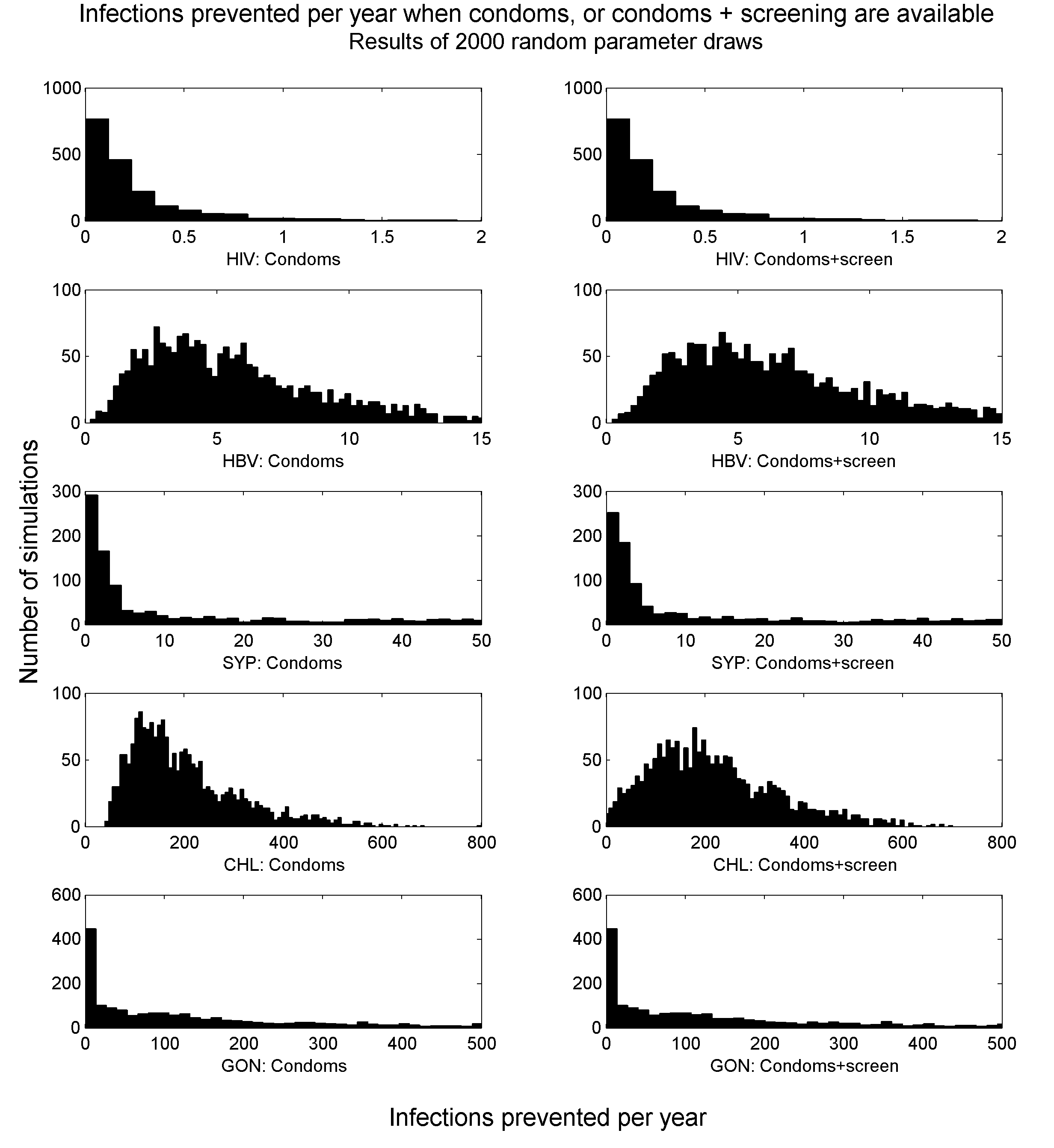

Supplement: S4 Fig — Histograms of HIV, Hepatitis B (HBV), syphilis (SYP), chlamydia (CHL) and gonorrhoea (GON) infections prevented per annum from 2000 simulations using random parameter draws, condom intervention (left) and condom with screening on arrival intervention (right). (TIF) [file pone.0144869.s004.tif]

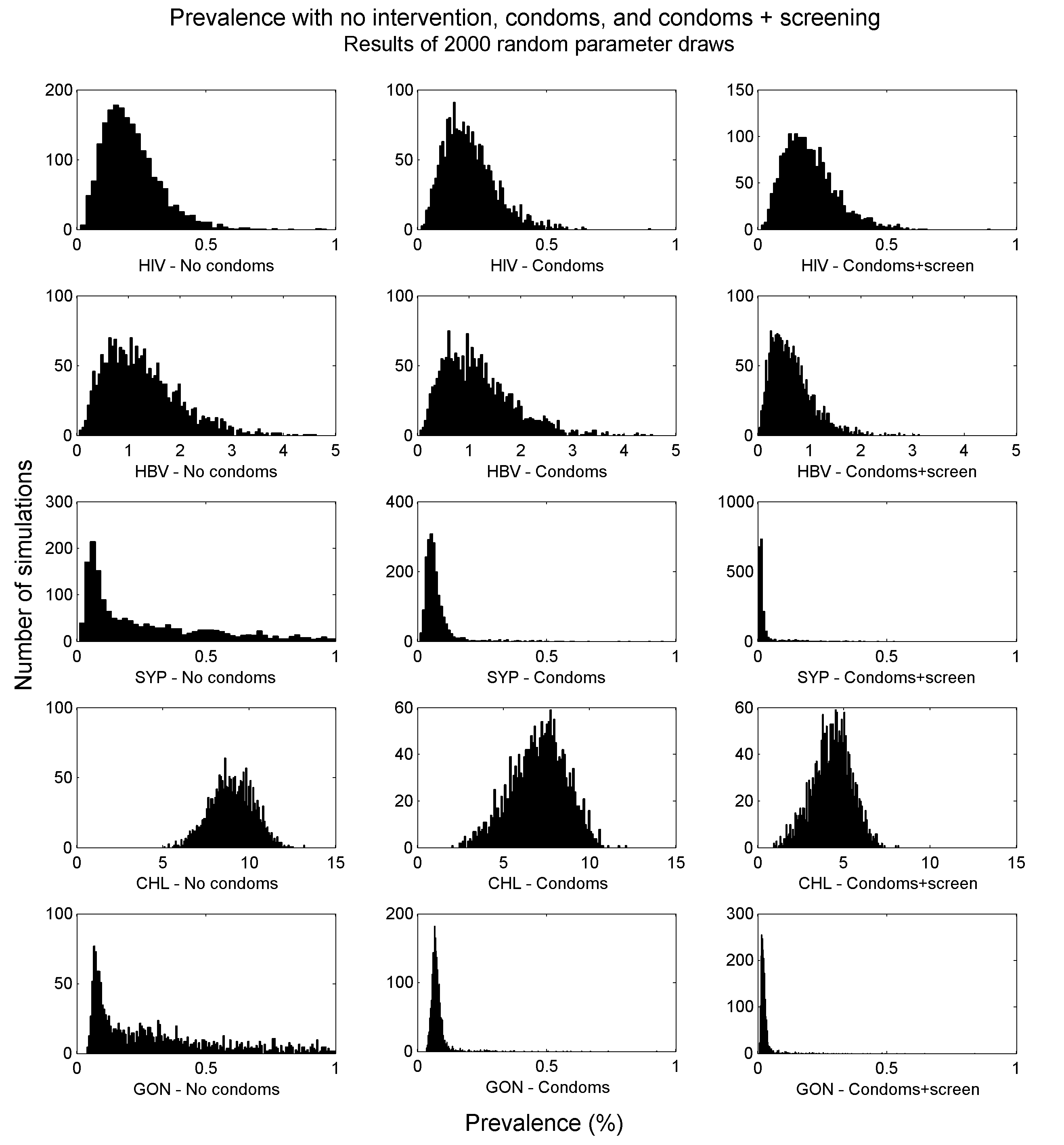

Supplement: S5 Fig — Histograms of HIV, Hepatitis B (HBV), syphilis (SYP), chlamydia (CHL) and gonorrhoea (GON) prevalence in prison using 2000 random parameter draws, before any interventions (left), after the introduction of condoms (middle), and after the introduction of condoms and a screening on arrival intervention (right). (TIF) [file pone.0144869.s005.tif]
