# Supplementary material for: Modelling the Impact of Condom Distribution on the Incidence and Prevalence of Sexually Transmitted Infections in an Adult Male Prison System
Source: PLoS One. 2015 Dec 14;10(12):e0144869. doi: 10.1371/journal.pone.0144869 (PMC4691199; doi:10.1371/journal.pone.0144869)
Supplement: S1 File — Detailed description of the equations used for each model; the apportioning of new arrivals in the hepatitis B model, dealing with immunity through the vaccination; and the distributions used for individual parameter uncertainties, the resulting distributions of number of infections prevented per year (with condoms and with condoms + screening on arrival), and the resulting distributions of the prevalence of each infection (without condoms, with condoms and with condoms + screening on arrival). (DOCX) [file pone.0144869.s006.docx]

**Supporting information**

Modelling the impact of condom distribution on the incidence and prevalence of sexually transmitted infections in an adult male prison system

Nick Scott, Emma McBryde, Amy Kirwan, Mark Stoové

**Model equations**

Let M be the 14x14 matrix with entries $m_{ij}$ equal to the proportion of prison i transferred into prison j each month when $i\neq j$, and entries $m_{ii}$ set to maintain equal prison populations:

$$m_{ii}\text{ }=\text{ }-\left(\text{total number of prisoners leaving prison }i\right)/\left(\text{population of prison }i\right).$$

Monthly data containing the complete number of prisoners transferred between each of the 14 prisons were obtained for the period 1 July 2012 to 31 June 2013, and the average monthly transfers used to populate the matrix M. Let $S=(S_{1 },\ldots,S_{14})$ with $S_{i}$ the number of susceptibles in prison *i*, and let $S\underline{M}\left[i\right]$ represent the ith component of the vector $S\underline{M}$. Then $S\underline{M}\left[i\right]$ is the net change in the number of susceptible prisoners in prison i due to transfers (both in and out), assuming transfers are independent of STI and sexual behaviour status. Note that $-S_{i}m_{ii}$ is the number of susceptibles transferred out of prison i, and $S\underline{M}\left[i\right]-S_{i}m_{ii}$ is the number of prisoners transferred into prison i. Analogous notation has been used for other compartments.

The spread of each disease in each prison (i=1,…,14) was modelled using the equations and forces of infections below. Where a screening intervention is available, a proportion ‘*screen’* of new arrivals (including transfers) are screened on arrival at a screening rate ρ. Parameter descriptions and estimates can be found in Table 1 and schematics, excluding transfers, are shown in Fig. 1-2.

**HIV, syphilis, chlamydia and gonorrhoea models, no screening on arrival**

$$\frac{dU_{i}}{dt}=\left(Ns_{i}+Nl_{i}\right)\times\left(1-sex\right)\times\left(1-Cinf\right)+\left(\frac{1}{\tau}+\eta\right)^{-1}V_{i}-\left(Ns_{i}+Nl_{i}\right)\frac{U_{i}}{P_{i}}+U\underline{M}\left[i\right]$$

$$\frac{dV_{i}}{dt}=\left(Ns_{i}+Nl_{i}\right)\times\left(1-sex\right)\times Cinf-\left(\frac{1}{\tau}+\eta\right)^{-1}V_{i}-\left(Ns_{i}+Nl_{i}\right)\frac{V_{i}}{P_{i}}+V\underline{M}\left[i\right]$$

$$\begin{matrix} \frac{dS_{i}}{dt}=\left(Ns_{i}+Nl_{i}\right)\times sex\times\left[\left(1-Prior\right)\times\left(1-Cinf\right)+Prior\times\left(1-\max(Pinf,C\inf)\right)\right] \\ -\lambda S_{i}+\frac{1}{\eta}T_{i}-\left(Ns_{i}+Nl_{i}\right)\frac{S_{i}}{P_{i}}+S\underline{M}\left[i\right] \end{matrix}$$

$$\frac{dL_{i}}{dt}=\lambda S_{i}-\frac{1}{\text{γ}}L_{i}-\left(Ns_{i}+Nl_{i}\right)\frac{L_{i}}{P_{i}}+L\underline{M}\left[i\right]$$

$$\begin{matrix} \frac{dI_{i}}{dt}=\left(Ns_{i}+Nl_{i}\right)\times sex\times\left[\left(1-Prior\right)\times Cinf+Prior\times\max(Pinf,C\inf)\right] \\ +\frac{1}{\text{γ}}L_{i}-\tau I_{i}-\left(Ns_{i}+Nl_{i}\right)\frac{I_{i}}{P_{i}}+I\underline{M}\left[i\right] \end{matrix}$$

$$\frac{dT_{i}}{dt}=\tau I_{i}-\frac{1}{\eta}T_{i}-\left(Ns_{i}+Nl_{i}\right)\frac{T_{i}}{P_{i}}+T\underline{M}\left[i\right]$$

With force of infection:

$$\lambda=\beta\times acts\times\left(1-condom\right)\times\frac{I_{i}+T_{i}}{S_{i}+L_{i}+I_{i}+T_{i}}$$

For the HIV model, the force of infection was modified to account for suppression of infectiousness when on antiviral treatments:

**Hepatitis B model, no screening on arrival**

$$\begin{matrix} \frac{dU_{i}}{dt}=\left(Ns_{i}+Nl_{i}\right)\times\{\left(1-sex\right)\times\left(1-Cinf\right)+sex\times[\left(1-Prior\right)\times VacC+Prior\times VacP]\}+\left(\frac{1}{\tau}+\eta\right)^{-1}V_{i} \\ -\left(Ns_{i}+Nl_{i}\right)\frac{U_{i}}{P_{i}}+U\underline{M}\left[i\right] \end{matrix}$$

$$\frac{dV_{i}}{dt}=\left(Ns_{i}+Nl_{i}\right)\times\left(1-sex\right)\times Cinf-\left(\frac{1}{\tau}+\eta\right)^{-1}V_{i}-\left(Ns_{i}+Nl_{i}\right)\frac{V_{i}}{P_{i}}+V\underline{M}\left[i\right]$$

$$\begin{matrix} \frac{dS_{i}}{dt}=\left(Ns_{i}+Nl_{i}\right)\times sex \\ \times\left[\left(1-Prior\right)\times\left(1-VacC\right)\times\left(1-Cinf\right)+Prior\times\left(1-VacP\right)\times\left(1-\max(Pinf,C\inf)\right)\right] \\ -\lambda S_{i}+\frac{1}{\eta}T_{i}-\left(Ns_{i}+Nl_{i}\right)\frac{S_{i}}{P_{i}}+S\underline{M}\left[i\right] \end{matrix}$$

$$\frac{dL_{i}}{dt}=\lambda S_{i}-\frac{1}{\text{γ}}L_{i}-\left(Ns_{i}+Nl_{i}\right)\frac{L_{i}}{P_{i}}+L\underline{M}\left[i\right]$$

$$\begin{matrix} \frac{dI_{i}}{dt}=\left(Ns_{i}+Nl_{i}\right)\times sex\times\left[\left(1-Prior\right)\times\left(1-VacC\right)\times Cinf+Prior\times\left(1-VacP\right)\times\max(Pinf,C\inf)\right] \\ +\frac{1}{\text{γ}}L_{i}-\tau I_{i}-\left(Ns_{i}+Nl_{i}\right)\frac{I_{i}}{P_{i}}+I\underline{M}\left[i\right] \end{matrix}$$

$$\frac{dT_{i}}{dt}=\tau I_{i}-\frac{1}{\eta}T_{i}-\left(Ns_{i}+Nl_{i}\right)\frac{T_{i}}{P_{i}}+T\underline{M}\left[i\right]$$

With force of infection:

$$\lambda=\beta\times acts\times[\left(Prior\times\left(1-VacP\right)+\left(1-Prior\right)\times\left(1-VacC\right)\right]\times\left(1-condom\right)\times\frac{I_{i}+T_{i}}{S_{i}+L_{i}+I_{i}+T_{i}}$$

**HIV, syphilis, chlamydia and gonorrhoea models, screening on arrival**

$$\begin{matrix} \frac{dU_{i}}{dt}=\left(Ns_{i}+Nl_{i}\right)\times\left(1-screen\right)\times\left(1-sex\right)\times\left(1-Cinf\right)+\rho U_{i}^{'}+\rho\frac{1}{\eta}V_{i}^{'}+\left(\frac{1}{\tau}+\eta\right)^{-1}V_{i} \\ -\left(Ns_{i}+Nl_{i}\right)\frac{U_{i}}{P_{i}}+\left(1-screen\right)\times\left(U\underline{M}\left[i\right]-U_{i}m_{ii}\right)-U_{i}m_{ii} \end{matrix}$$

$$\begin{matrix} \frac{dV_{i}}{dt}=\left(Ns_{i}+Nl_{i}\right)\times\left(1-screen\right)\times\left(1-sex\right)\times Cinf-\left(\frac{1}{\tau}+\eta\right)^{-1}V_{i}-\left(Ns_{i}+Nl_{i}\right)\frac{V_{i}}{P_{i}} \\ +\left(1-screen\right)\times\left(V\underline{M}\left[i\right]-V_{i}m_{ii}\right)-V_{i}m_{ii} \end{matrix}$$

$$\begin{matrix} \frac{dS_{i}}{dt}=\left(Ns_{i}+Nl_{i}\right)\times sex\times\left(1-screen\right)\times\left[\left(1-Prior\right)\times\left(1-Cinf\right)+Prior\times\left(1-\max(Pinf,C\inf)\right)\right] \\ +\rho S_{i}^{'}-\lambda S_{i}+\frac{1}{\eta}T_{i}-\left(Ns_{i}+Nl_{i}\right)\frac{S_{i}}{P_{i}}+\left(1-screen\right)\times\left(S\underline{M}\left[i\right]-S_{i}m_{ii}\right)-S_{i}m_{ii} \end{matrix}$$

$$\frac{dL_{i}}{dt}=\frac{\rho\omega}{\gamma}L_{i}^{'}+\lambda S_{i}-\frac{1}{\text{γ}}L_{i}-\left(Ns_{i}+Nl_{i}\right)\frac{L_{i}}{P_{i}}+\left(1-screen\right)\times\left(L\underline{M}\left[i\right]-L_{i}m_{ii}\right)-Lm_{ii}$$

$$\begin{matrix} \frac{dI_{i}}{dt}=\left(Ns_{i}+Nl_{i}\right)\times\left(1-screen\right)\times sex\times\left[\left(1-Prior\right)\times Cinf+Prior\times\max(Pinf,C\inf)\right] \\ +\frac{1}{\text{γ}}L_{i}-\tau I_{i}-\left(Ns_{i}+Nl_{i}\right)\frac{I_{i}}{P_{i}}+(1-screen\times\left(I\underline{M}\left[i\right]-I_{i}m_{ii}\right)-I_{i}m_{ii} \end{matrix}$$

$$\frac{dT_{i}}{dt}=\rho T_{i}^{'}+\rho\left(1-\frac{\omega}{\gamma}\right)L_{i}^{'}+\rho I_{i}^{'}+\tau I_{i}-\frac{1}{\eta}T_{i}-\left(Ns_{i}+Nl_{i}\right)\frac{T_{i}}{P_{i}}+\left(1-screen\right)\times\left(T\underline{M}\left[i\right]-T_{i}m_{ii}\right)-T_{i}m_{ii}$$

$$\begin{matrix} \frac{dU_{i}^{'}}{dt}=\left(Ns_{i}+Nl_{i}\right)\times screen\times\left(1-sex\right)\times\left(1-Cinf\right)-\rho U_{i}^{'}+\left(\frac{1}{\tau}+\eta\right)^{-1}V_{i}^{'}-\left(Ns_{i}+Nl_{i}\right)\frac{U_{i}^{'}}{P_{i}} \\ +screen\times\left(U\underline{M}\left[i\right]-U_{i}m_{ii}\right)+U'\underline{M}\left[i\right] \end{matrix}$$

$$\begin{matrix} \frac{dV_{i}^{'}}{dt}=\left(Ns_{i}+Nl_{i}\right)\times screen\times\left(1-sex\right)\times Cinf-\rho\frac{1}{\eta}V_{i}^{'}-\left(\frac{1}{\tau}+\eta\right)^{-1}V_{i}^{'}-\left(Ns_{i}+Nl_{i}\right)\frac{V_{i}^{'}}{P_{i}} \\ +screen\times\left(V\underline{M}\left[i\right]-Vm_{ii}\right)+V'\underline{M}\left[i\right] \end{matrix}$$

$$\begin{matrix} \frac{dS_{i}^{'}}{dt}=\left(Ns_{i}+Nl_{i}\right)\times sex\times screen\times\left[\left(1-Prior\right)\times\left(1-Cinf\right)+Prior\times\left(1-\max(Pinf,C\inf)\right)\right] \\ -\rho S_{i}^{'}-\lambda S_{i}^{'}+\frac{1}{\eta}T_{i}^{'}-\left(Ns_{i}+Nl_{i}\right)\frac{S_{i}^{'}}{P_{i}}+screen\times\left(S\underline{M}\left[i\right]-S_{i}m_{ii}\right)+S'\underline{M}\left[i\right] \end{matrix}$$

$$\frac{dL_{i}^{'}}{dt}=-\rho L_{i}^{'}+\lambda S_{i}^{'}-\frac{1}{\text{γ}}L_{i}^{'}-\left(Ns_{i}+Nl_{i}\right)\frac{L_{i}^{'}}{P_{i}}+screen\times\left(L\underline{M}\left[i\right]-L_{i}m_{ii}\right)+L'\underline{M}\left[i\right]$$

$$\begin{matrix} \frac{dI_{i}^{'}}{dt}=\left(Ns_{i}+Nl_{i}\right)\times screen\times sex\times\left[\left(1-Prior\right)\times Cinf+Prior\times\max(Pinf,C\inf)\right]-\rho I_{i}^{'}+\frac{1}{\text{γ}}L_{i}^{'}-\tau I_{i}^{'} \\ -\left(Ns_{i}+Nl_{i}\right)\frac{I_{i}^{'}}{P_{i}}+screen\times\left(I\underline{M}\left[i\right]-I_{i}m_{ii}\right)+I'\underline{M}\left[i\right] \end{matrix}$$

$$\frac{dT_{i}^{'}}{dt}=-\rho T_{i}^{'}+\tau I_{i}^{'}-\frac{1}{\eta}T_{i}^{'}-\left(Ns_{i}+Nl_{i}\right)\frac{T_{i}^{'}}{P_{i}}+screen\times\left(T\underline{M}\left[i\right]-T_{i}m_{ii}\right)+T'\underline{M}\left[i\right]$$

With force of infection:

$$\lambda=\beta\times acts\times\left(1-condom\right)\times\frac{I_{i}+T_{i}+I_{i}^{'}+T_{i}^{'}}{S_{i}+L_{i}+I_{i}+T_{i}+S_{i}^{'}+L_{i}^{'}+I_{i}^{'}+T_{i}^{'}}$$

For the HIV model, the force of infection was modified to account for suppression of infectiousness when on antiviral treatments:

**Hepatitis B model, screening on arrival**

$$\begin{matrix} \frac{dU_{i}}{dt}=\left(Ns_{i}+Nl_{i}\right)\times\left(1-screen\right)\times\left\{\left(1-sex\right)\times\left(1-Cinf\right)+sex\times\left[\left(1-Prior\right)\times VacC+Prior\times VacP\right]\right\} \\ +\rho U_{i}^{'}+\rho\frac{1}{\eta}V_{i}^{'}+\left(\frac{1}{\tau}+\eta\right)^{-1}V_{i}-\left(Ns_{i}+Nl_{i}\right)\frac{U_{i}}{P_{i}}+\left(1-screen\right)\times U\underline{M}\left[i\right] \end{matrix}$$

$$\frac{dV_{i}}{dt}=\left(Ns_{i}+Nl_{i}\right)\times\left(1-screen\right)\times\left(1-sex\right)\times Cinf-\left(\frac{1}{\tau}+\eta\right)^{-1}V_{i}-\left(Ns_{i}+Nl_{i}\right)\frac{V_{i}}{P_{i}}+\left(1-screen\right)\times V\underline{M}\left[i\right]$$

$$\begin{matrix} \frac{dS_{i}}{dt}=\left(Ns_{i}+Nl_{i}\right)\times sex\times\left(1-screen\right)\times[\left(1-Prior\right)\times\left(1-VacC\right)\times\left(1-Cinf\right) \\ +Prior\times\left(1-VacP\right)\times\left(1-\max(Pinf,C\inf)\right)]+\rho S_{i}^{'}-\lambda S_{i} \\ +\frac{1}{\eta}T_{i}-\left(Ns_{i}+Nl_{i}\right)\frac{S_{i}}{P_{i}}+\left(1-screen\right)\times S\underline{M}\left[i\right] \end{matrix}$$

$$\frac{dL_{i}}{dt}=\frac{\rho\omega}{\gamma}L_{i}^{'}+\lambda S_{i}-\frac{1}{\text{γ}}L_{i}-\left(Ns_{i}+Nl_{i}\right)\frac{L_{i}}{P_{i}}+\left(1-screen\right)\times L\underline{M}\left[i\right]$$

$$\begin{matrix} \frac{dI_{i}}{dt}=\left(Ns_{i}+Nl_{i}\right)\times\left(1-screen\right)\times sex \\ \times\left[\left(1-Prior\right)\times\left(1-VacC\right)\times Cinf+Prior\times\left(1-VacP\right)\times\max(Pinf,C\inf)\right] \\ +\frac{1}{\text{γ}}L_{i}-\tau I_{i}-\left(Ns_{i}+Nl_{i}\right)\frac{I_{i}}{P_{i}}+\left(1-screen\right)\times I\underline{M}\left[i\right] \end{matrix}$$

$$\frac{dT_{i}}{dt}=\rho T_{i}^{'}+\rho\left(1-\frac{\omega}{\gamma}\right)L_{i}^{'}+\rho I_{i}^{'}+\tau I_{i}-\frac{1}{\eta}T_{i}-\left(Ns_{i}+Nl_{i}\right)\frac{T_{i}}{P_{i}}+\left(1-screen\right)\times T\underline{M}\left[i\right]$$

$$\begin{matrix} \frac{dU_{i}^{'}}{dt}=\left(Ns_{i}+Nl_{i}\right)\times screen\times\left\{\left(1-sex\right)\times\left(1-Cinf\right)+sex\times\left[\left(1-Prior\right)\times VacC+Prior\times VacP\right]\right\} \\ -\rho U_{i}^{'}+\left(\frac{1}{\tau}+\eta\right)^{-1}V_{i}^{'}-\left(Ns_{i}+Nl_{i}\right)\frac{U_{i}^{'}}{P_{i}}+screen\times U\underline{M}\left[i\right]+U'\underline{M}\left[i\right] \end{matrix}$$

$$\begin{matrix} \frac{dV_{i}^{'}}{dt}=\left(Ns_{i}+Nl_{i}\right)\times screen\times\left(1-sex\right)\times Cinf-\rho\frac{1}{\eta}V_{i}^{'}-\left(\frac{1}{\tau}+\eta\right)^{-1}V_{i}^{'}-\left(Ns_{i}+Nl_{i}\right)\frac{V_{i}^{'}}{P_{i}} \\ +screen\times V\underline{M}\left[i\right]+V'\underline{M}\left[i\right] \end{matrix}$$

$$\begin{matrix} \frac{dS_{i}^{'}}{dt}=\left(Ns_{i}+Nl_{i}\right)\times sex\times screen \\ \times\left[\left(1-Prior\right)\times\left(1-VacC\right)\times\left(1-Cinf\right)+Prior\times\left(1-VacP\right)\times\left(1-\max(Pinf,C\inf)\right)\right] \\ -\rho S_{i}^{'}-\lambda S_{i}^{'}+\frac{1}{\eta}T_{i}^{'}-\left(Ns_{i}+Nl_{i}\right)\frac{S_{i}^{'}}{P_{i}}+screen\times S\underline{M}\left[i\right]+S'\underline{M}\left[i\right] \end{matrix}$$

$$\frac{dL_{i}^{'}}{dt}=-\rho L_{i}^{'}+\lambda S_{i}^{'}-\frac{1}{\text{γ}}L_{i}^{'}-\left(Ns_{i}+Nl_{i}\right)\frac{L_{i}^{'}}{P_{i}}+screen\times L\underline{M}\left[i\right]+L'\underline{M}\left[i\right]$$

$$\begin{matrix} \frac{dI_{i}^{'}}{dt}=\left(Ns_{i}+Nl_{i}\right)\times screen\times sex \\ \times\left[\left(1-Prior\right)\times\left(1-VacC\right)\times Cinf+Prior\times\left(1-VacP\right)\times\max(Pinf,C\inf)\right] \\ -\rho I_{i}^{'}+\frac{1}{\text{γ}}L_{i}^{'}-\tau I_{i}^{'}-\left(Ns_{i}+Nl_{i}\right)\frac{I_{i}^{'}}{P_{i}}+screen\times I\underline{M}\left[i\right]+I'\underline{M}\left[i\right] \end{matrix}$$

$$\frac{dT_{i}^{'}}{dt}=-\rho T_{i}^{'}+\tau I_{i}^{'}-\frac{1}{\eta}T_{i}^{'}-\left(Ns_{i}+Nl_{i}\right)\frac{T_{i}^{'}}{P_{i}}+screen\times T\underline{M}\left[i\right]+T'\underline{M}\left[i\right]$$

With force of infection:

$$\begin{matrix} \lambda=\beta\times acts\times[\left(Prior\times\left(1-VacP\right)+\left(1-Prior\right)\times\left(1-VacC\right)\right]\times\left(1-condom\right) \\ \times\frac{I_{i}+T_{i}+I_{i}^{'}+T_{i}^{'}}{S_{i}+L_{i}+I_{i}+T_{i}+S_{i}^{'}+L_{i}^{'}+I_{i}^{'}+T_{i}^{'}} \end{matrix}$$

**Apportioning new arrivals in the Hepatitis B model**


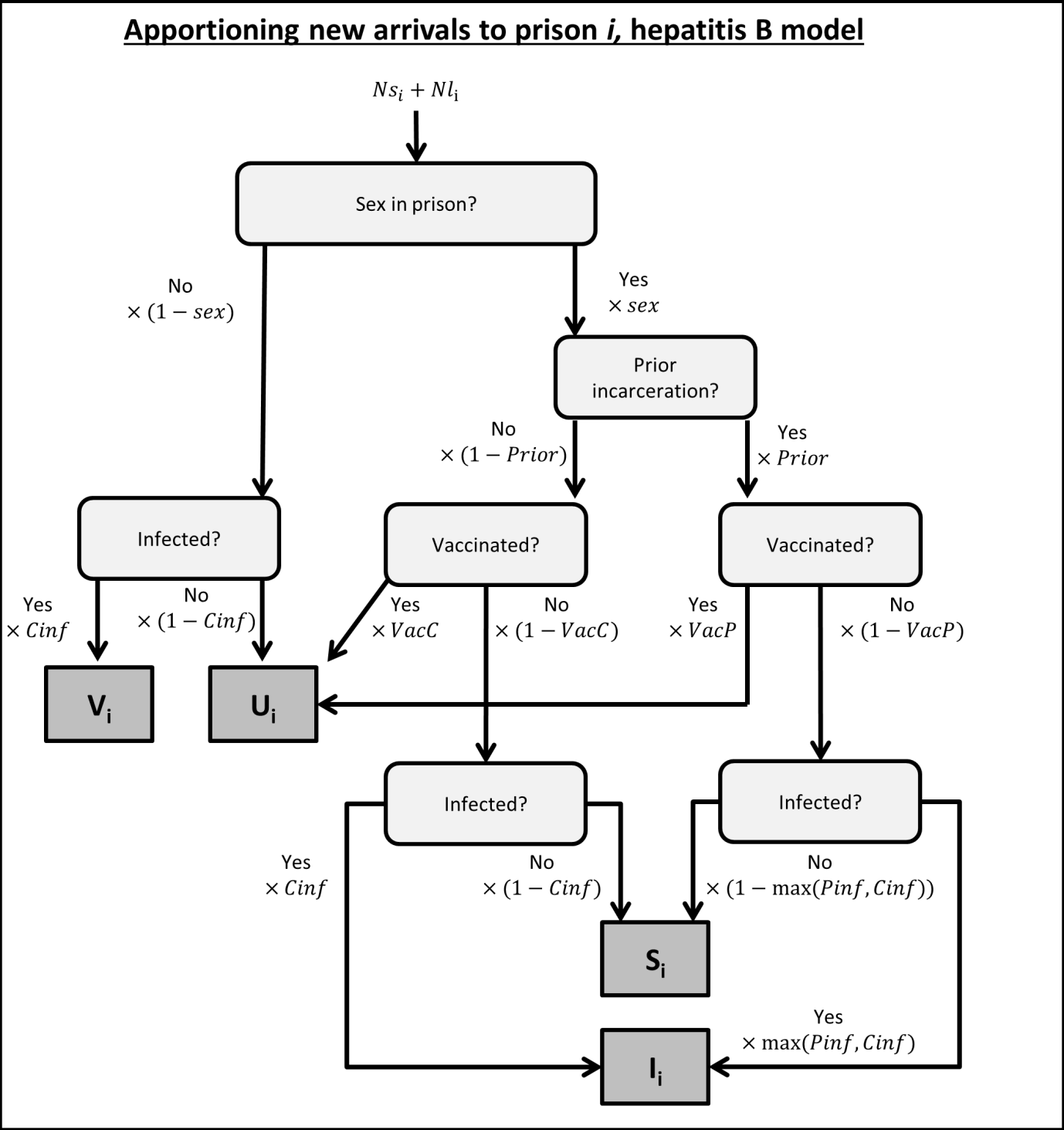


S1 Fig. Entry into the hepatitis B model. For the hepatitis B model, each month Ns_i_+Nl_i_ new prisoners arrive in prison i and are apportioned to compartments according to sexual activity in prison, prior incarceration status vaccination status and community or prison infection prevalence.

**Uncertainty analysis**

Table S1 shows the Beta distributions that were used to estimate the uncertainty around the parameters Sex, Used, Condom, Prior, ρ, screen, VacHB, Cinf, β, τ, γ and ω. The corresponding distributions are plotted in S2 Fig and S3 Fig.

The results of the Monte Carlo uncertainty analysis using 2000 random parameter draws from these distributions are shown in S4 Fig and S5 Fig.

**Table S1. Parameter uncertainty distributions.** The uncertainties of individual parameters were parameterised as Beta probability distributions with parameters b_1_ and b_2_.

|  | | **b_1_** | **b_2_** | **b_1_** | **b_2_** | **b_1_** | **b_2_** | **b_1_** | **b_2_** | **b_1_** | **b_2_** |
| --- | --- | --- | --- | --- | --- | --- | --- | --- | --- | --- | --- |
| **Parameter** | | **HIV** | | **Hepatitis B** | | **Syphilis** | | **Chlamydia** | | **Gonorrhoea** | |
| Sex | Proportion of sexually active prisoners | 2.86 | 28.90 | 3.55 | 35.89 | 3.55 | 35.89 | 3.55 | 35.89 | 3.55 | 35.89 |
| Used | Proportion of distributed condoms used for sex | 38.00 | 57.00 | 38.00 | 57.00 | 38.00 | 57.00 | 38.00 | 57.00 | 38.00 | 57.00 |
| Condom | Proportion of sexual acts using condoms (when available) | 51.40 | 47.44 | 51.40 | 47.44 | 51.40 | 47.44 | 51.40 | 47.44 | 51.40 | 47.44 |
| Prior | Proportion of prisoners with prior incarceration | 47.44 | 51.40 | 47.44 | 51.40 | 47.44 | 51.40 | 47.44 | 51.40 | 47.44 | 51.40 |
| ρ | Screening rate | 3.50* | 31.50* | 3.50* | 31.50* | 3.50* | 31.50* | 3.50* | 31.50* | 3.50* | 31.50* |
| screen | Proportion who are screened | 17.10 | 0.90 | 17.10 | 0.90 | 17.10 | 0.90 | 17.10 | 0.90 | 17.10 | 0.90 |
| VacHB  (Com.) | HBV vaccination prevalence (Community) | - | - | 24.90 | 58.10 | - | - | - | - | - | - |
| VacHB (Prison) | HBV Vaccination prevalence (Prison) | - | - | 49.50 | 49.50 | - | - | - | - | - | - |
| Cinf | Community infection prevalence | 4.00 | 1991.00 | 2.82 | 163.29 | 6.70 | 2585.50 | 58.40 | 1439.70 | 26.90 | 5145.10 |
| β | Risk of transmission per sexual act | 1.92 | 135.12 | 45.50 | 604.50 | 7.50 | 42.50 | 31.50 | 58.50 | 14.88 | 52.76 |
| τ | Effective detection and treatment rate per month | 29.14 | 494.95 | 58.80 | 676.20 | 49.50 | 49.50 | 2.28 | 26.16 | 89.90* | 809.10* |
| γ | Duration of latency (months) | 4.44 | 13.31 | 1439.40* | 959.60* | 13.31 | 4.44 | 8.57 | 13.99 | 4.44 | 13.31 |
| ω | Window period | 13.31 | 4.44 | 89.90* | 809.10* | 8.41 | 14.02 | 4.44 | 13.31 | 1.76 | 9.99 |

*Indicates that Beta distributions were scaled to have support on the interval [0,10] rather than [0,1].


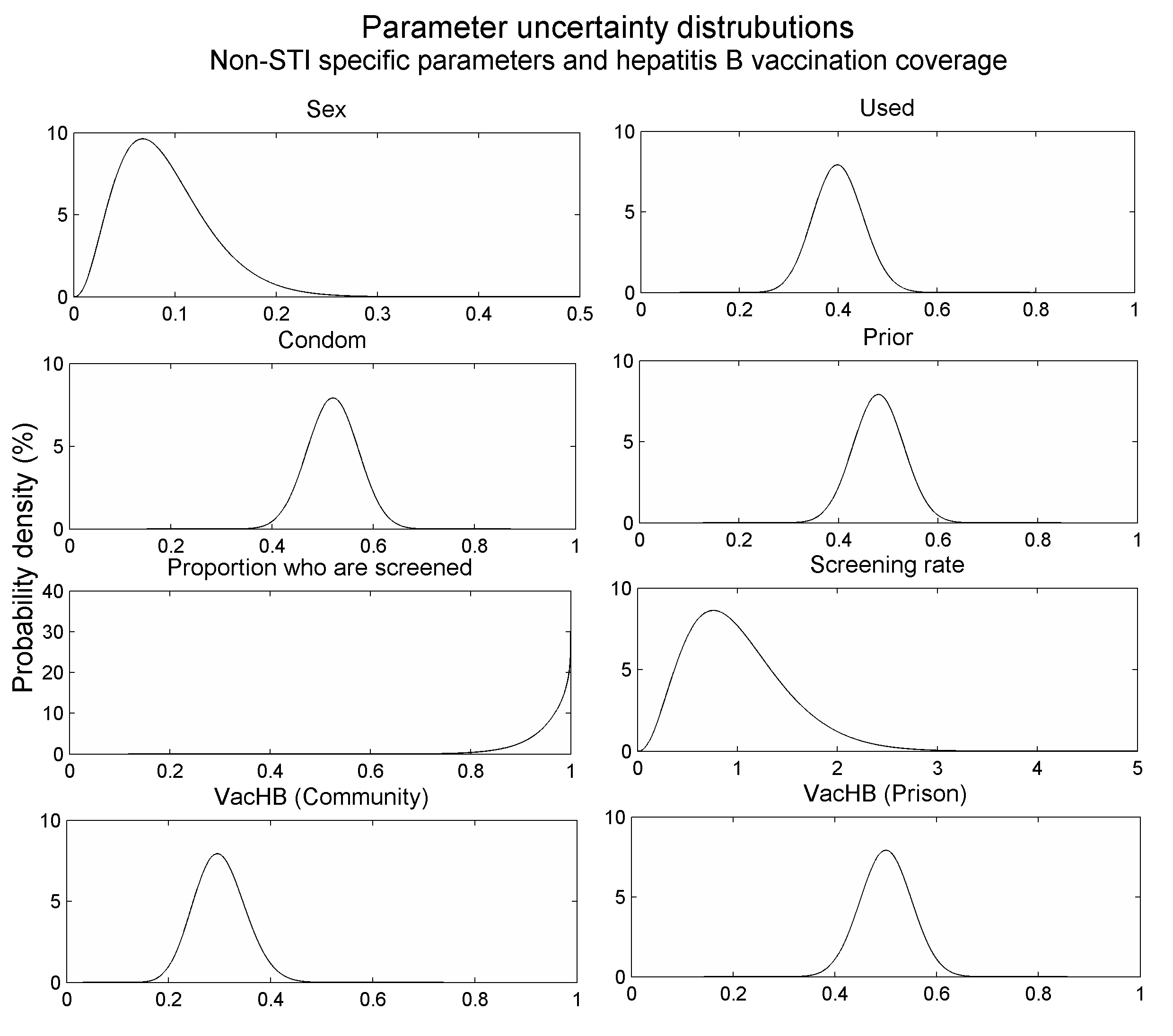


**S2 Fig. Uncertainty distributions for non-STI specific parameters and hepatitis B vaccination coverage parameters.** Assumed uncertainty of parameters for: the proportion of prisoners who are sexually active (Sex); the proportion of condoms used for sex (Used); the proportion of sexual acts that use condoms when available (Condom); the proportion of prisoners with a history of incarceration (Prior); the proportion of prisoners who are screened on arrival when the intervention is available and the rate they are screened at (in months); and the prevalence of hepatitis B vaccination in the community [VacHB (Community)] and in prison [VacHV (Prison)].


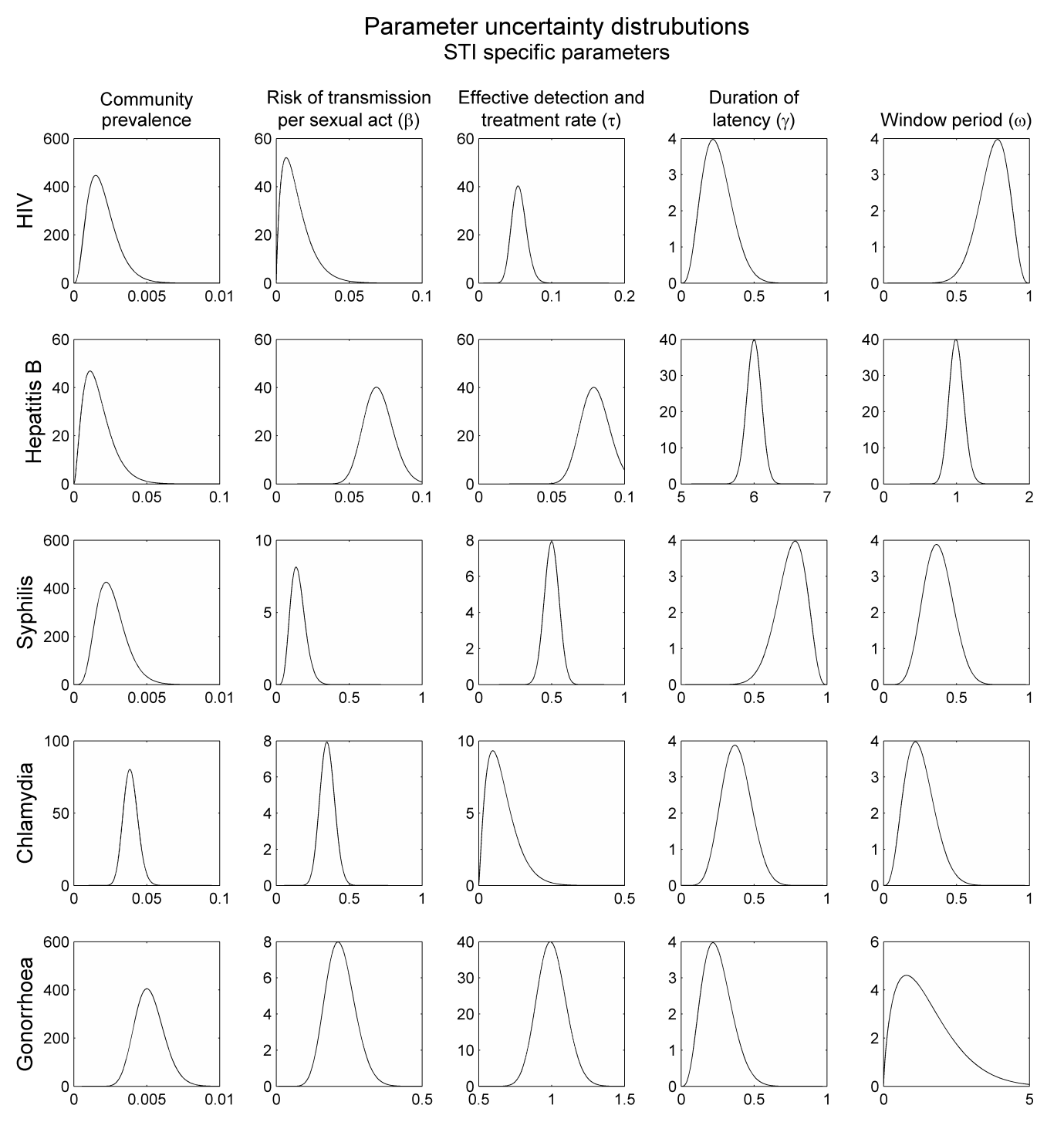


**S3 Fig. Uncertainty distributions for STI specific parameters.** Assumed uncertainty of parameters for the community STI prevalence (proportion of community infected), the risk of transmission per sexual act, the effective detection and treatment rate (in months), the duration of latency (in months) and the window period (in months).

***
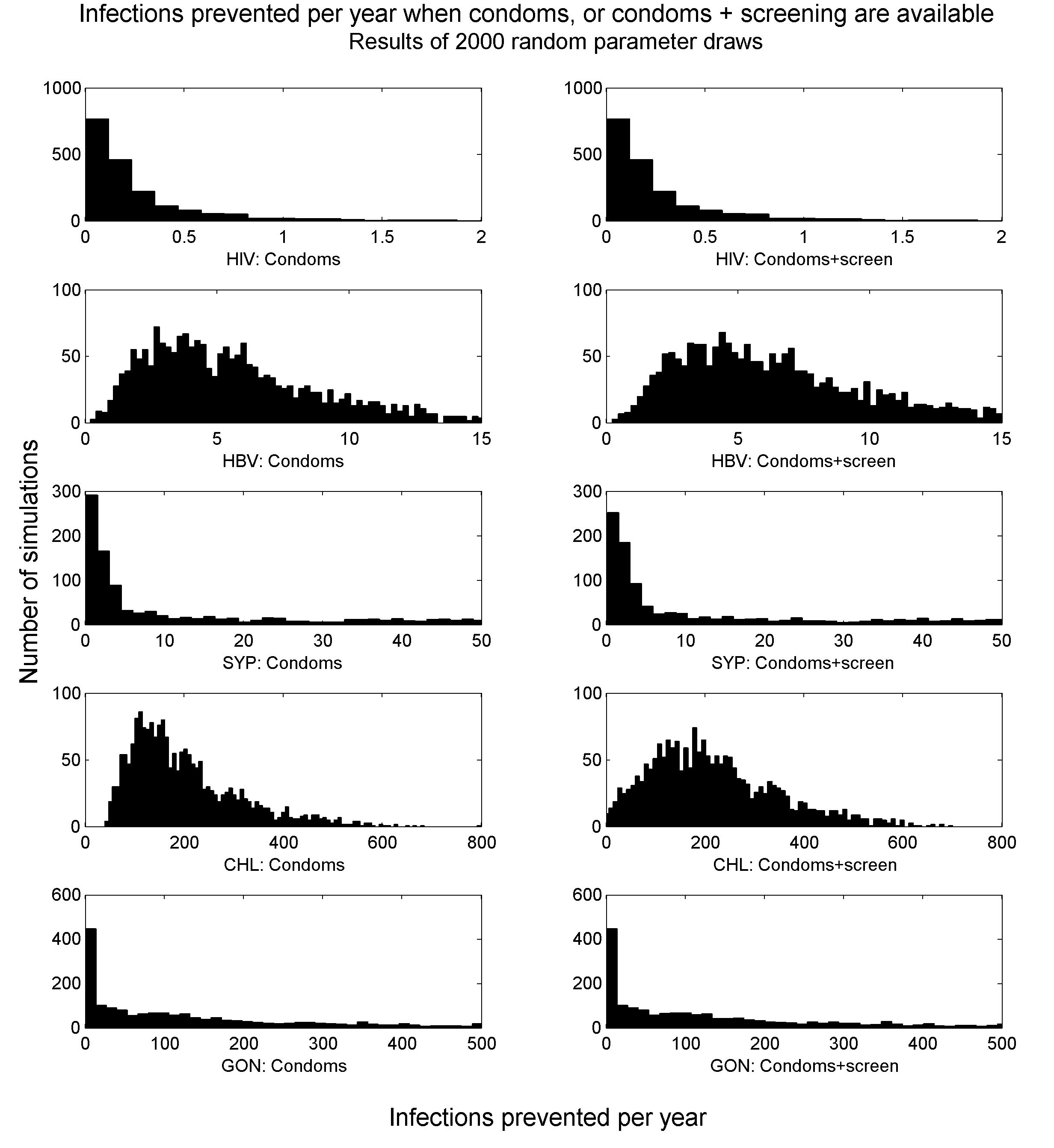
***

**S4 Fig. Uncertainty analysis infections averted.** Histograms of HIV, Hepatitis B (HBV), syphilis (SYP), chlamydia (CHL) and gonorrhoea (GON) infections prevented per annum from 2000 simulations using random parameter draws, condom intervention (left) and condom with screening on arrival intervention (right).


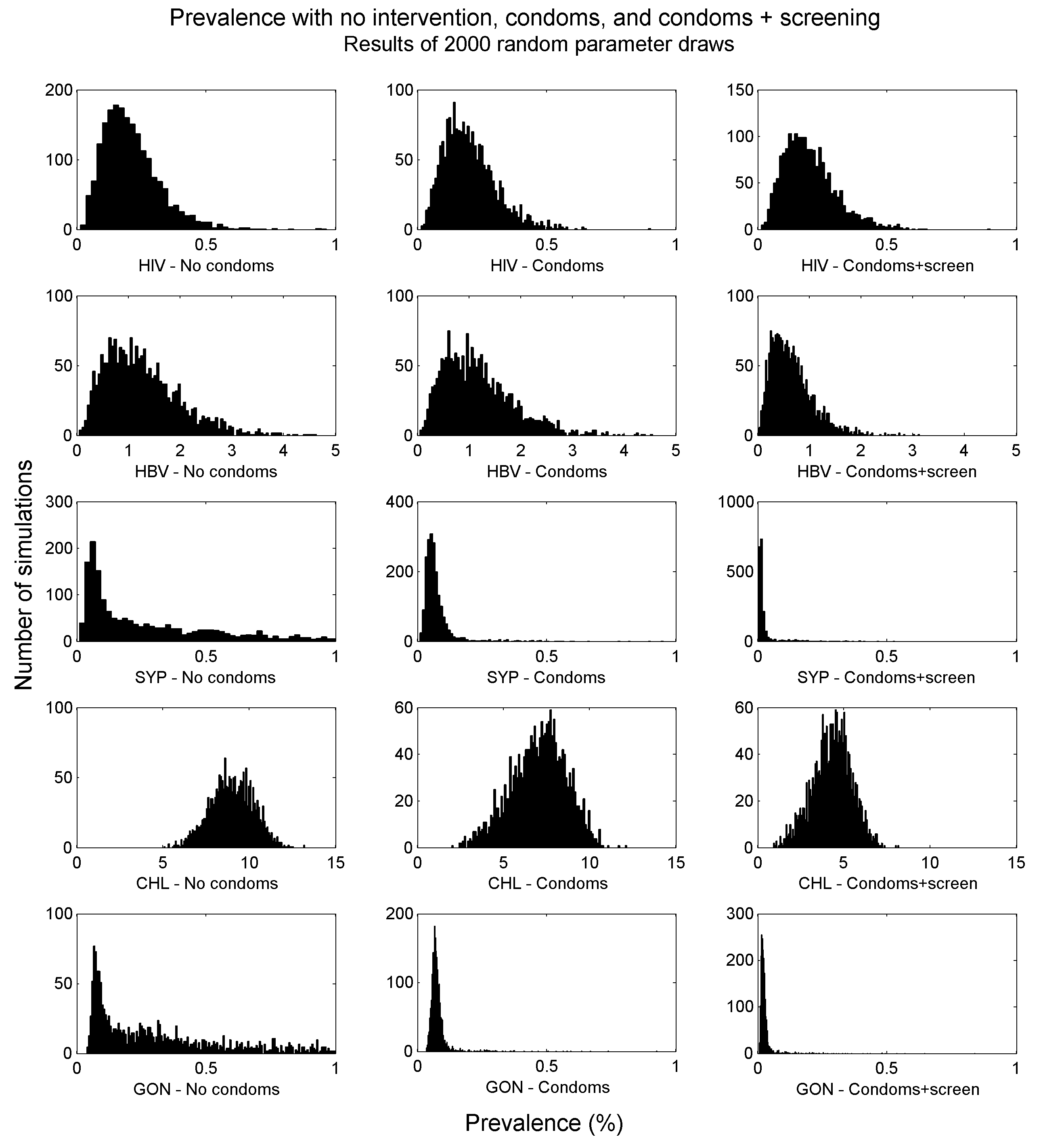


S5 Fig. Uncertainty analysis prevalence results. Histograms of HIV, Hepatitis B (HBV), syphilis (SYP), chlamydia (CHL) and gonorrhoea (GON) prevalence in prison using 2000 random parameter draws, before any interventions (left), after the introduction of condoms (middle), and after the introduction of condoms and a screening on arrival intervention (right).
