# Supplementary material for: Modelling the Impact of Condom Distribution on the Incidence and Prevalence of Sexually Transmitted Infections in an Adult Male Prison System
Source: PLoS One. 2015 Dec 14;10(12):e0144869. doi: 10.1371/journal.pone.0144869 (PMC4691199; doi:10.1371/journal.pone.0144869)
Supplement: S1 Table — The uncertainties of individual parameters were parameterised as Beta probability distributions with parameters b1 and b2. (DOCX) [file pone.0144869.s007.docx]

**S1 Table. Parameter uncertainty distributions.** The uncertainties of individual parameters were parameterised as Beta probability distributions with parameters b_1_ and b_2._

|  | | **b_1_** | **b_2_** | **b_1_** | **b_2_** | **b_1_** | **b_2_** | **b_1_** | **b_2_** | **b_1_** | **b_2_** |
| --- | --- | --- | --- | --- | --- | --- | --- | --- | --- | --- | --- |
| **Parameter** | | **HIV** | | **Hepatitis B** | | **Syphilis** | | **Chlamydia** | | **Gonorrhoea** | |
| Sex | Proportion of sexually active prisoners | 2.86 | 28.90 | 3.55 | 35.89 | 3.55 | 35.89 | 3.55 | 35.89 | 3.55 | 35.89 |
| Used | Proportion of distributed condoms used for sex | 38.00 | 57.00 | 38.00 | 57.00 | 38.00 | 57.00 | 38.00 | 57.00 | 38.00 | 57.00 |
| Condom | Proportion of sexual acts using condoms (when available) | 51.40 | 47.44 | 51.40 | 47.44 | 51.40 | 47.44 | 51.40 | 47.44 | 51.40 | 47.44 |
| Prior | Proportion of prisoners with prior incarceration | 47.44 | 51.40 | 47.44 | 51.40 | 47.44 | 51.40 | 47.44 | 51.40 | 47.44 | 51.40 |
| ρ | Screening rate | 3.50* | 31.50* | 3.50* | 31.50* | 3.50* | 31.50* | 3.50* | 31.50* | 3.50* | 31.50* |
| screen | Proportion who are screened | 17.10 | 0.90 | 17.10 | 0.90 | 17.10 | 0.90 | 17.10 | 0.90 | 17.10 | 0.90 |
| VacHB  (Com.) | HBV vaccination prevalence (Community) | - | - | 24.90 | 58.10 | - | - | - | - | - | - |
| VacHB (Prison) | HBV Vaccination prevalence (Prison) | - | - | 49.50 | 49.50 | - | - | - | - | - | - |
| Cinf | Community infection prevalence | 4.00 | 1991.00 | 2.82 | 163.29 | 6.70 | 2585.50 | 58.40 | 1439.70 | 26.90 | 5145.10 |
| β | Risk of transmission per sexual act | 1.92 | 135.12 | 45.50 | 604.50 | 7.50 | 42.50 | 31.50 | 58.50 | 14.88 | 52.76 |
| τ | Effective detection and treatment rate per month | 29.14 | 494.95 | 58.80 | 676.20 | 49.50 | 49.50 | 2.28 | 26.16 | 89.90* | 809.10* |
| γ | Duration of latency (months) | 4.44 | 13.31 | 1439.40* | 959.60* | 13.31 | 4.44 | 8.57 | 13.99 | 4.44 | 13.31 |
| ω | Window period | 13.31 | 4.44 | 89.90* | 809.10* | 8.41 | 14.02 | 4.44 | 13.31 | 1.76 | 9.99 |

*Indicates that Beta distributions were scaled to have support on the interval [0,10] rather than [0,1].
